# Ligand-directed dibromophenyl benzoate chemistry for rapid and selective acylation of intracellular natural proteins†
†Electronic supplementary information (ESI) available: Figures for eDHFR labeling *in vitro* and in cells, HPLC analyses of the stabilities of the reagents, CPK models of the reagents, and synthetic methods. See DOI: 10.1039/c5sc00190k



**DOI:** 10.1039/c5sc00190k

**Published:** 2015-03-23

**Authors:** Yousuke Takaoka, Yuki Nishikawa, Yuki Hashimoto, Kenta Sasaki, Itaru Hamachi

**Affiliations:** a Department of Synthetic Chemistry and Biological Chemistry , Graduate School of Engineering Kyoto University , Katsura , Kyoto 615-8510 , Japan . Email: ihamachi@sbchem.kyoto-u.ac.jp ; Fax: +81-22-795-6557 ; Tel: +81-22-795-6557; b Core Research for Evolutional Science and Technology (CREST) , Japan Science and Technology Agency , 5 Sanbancho , Chiyoda-ku , Tokyo 102-0075 , Japan

## Abstract

A rapid and selective ligand-directed chemical reaction was developed for the acylation of proteins in living cells on the basis of ligand-directed chemistry. By fine tuning the reactivity and stability of the phenyl ester derivatives, we successfully identified *ortho*-dibromophenyl benzoate as the optimal reactive motif. It was sufficiently stable in an aqueous buffer, hydrolyzing less than 10% after 13 h of incubation, but reactive enough for efficient and selective protein labeling in living mammalian cells, as well as *in vitro* (referred to as ligand-directed dibromophenyl benzoate (LDBB) chemistry). Using this chemistry, various fluorophores can be tethered to the target protein directly, which allows fluorescence visualization of the labeled protein in live cells using different colored fluorophore groups (including coumarin, fluorescein and rhodamine). Furthermore, this labeling is applicable to not only an overexpressed protein (*E. coli* dihydrofolate reductase) but also endogenous human carbonic anhydrase II and XII under living cell conditions. LDBB chemistry is a new entry of ligand-directed protein labeling methods, and should be particularly useful for the imaging of natural proteins in living cells.

## Abstract 2

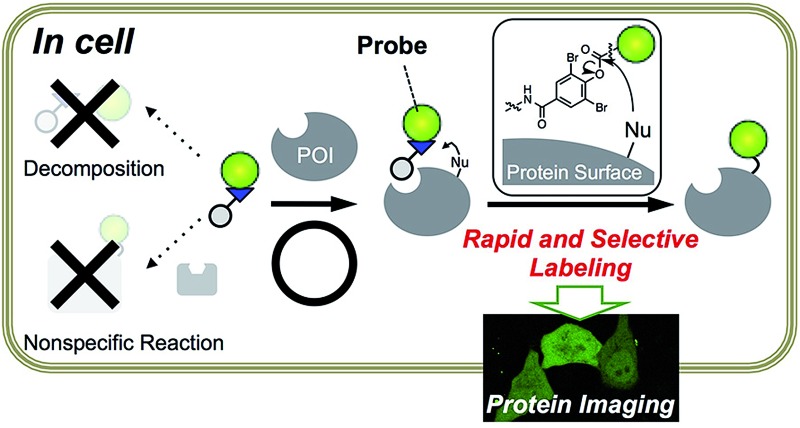
A rapid and selective protein labeling method, LDBB chemistry is a useful tool for natural protein imaging in living cells.

## Introduction

Natural protein labeling by synthetic fluorophores in living cells is powerful for investigating protein localization, quantification or function under native conditions, owing to the small size, variety of colors or micro-environment sensitivity of fluorophores, which are distinct from fluorescent proteins.^1–3^ We have recently proposed ligand-directed (LD) chemistry as a new strategy for the non-invasive and specific labeling of endogenous proteins in live cells. In particular, ligand-directed tosyl (LDT) chemistry allowed for covalent labeling of natural proteins with synthetic fluorophores, by which we constructed a semisynthetic biosensor inside living cells.^4^ Fluorophore tethering to natural membrane-bound proteins was also carried out using recently developed ligand-directed acyl imidazole (LDAI) chemistry.^5^ Fenical and co-workers independently reported another type of LD chemistry using a phenyl-ester (LDPE) for the identification and visualization of a natural product target protein.^6^ Despite their high selectivity, these methods often suffer from slow reaction rates (about 10 h of reaction time) and low labeling efficiency, which hampers the fluorescent imaging of intracellular proteins.^4,5^ On the other hand, simply increasing the reactivity of the labeling reagents to accelerate the reaction would result in non-specific reactions toward non-target proteins and/or nonproductive hydrolysis of the reagents. Clearly, for the selective imaging of “intracellular” protein using LD chemistry, a balanced reactive motif equipped with the two conflicting capabilities, selectivity and fast kinetics, is required.^7^

**Fig. 1:**
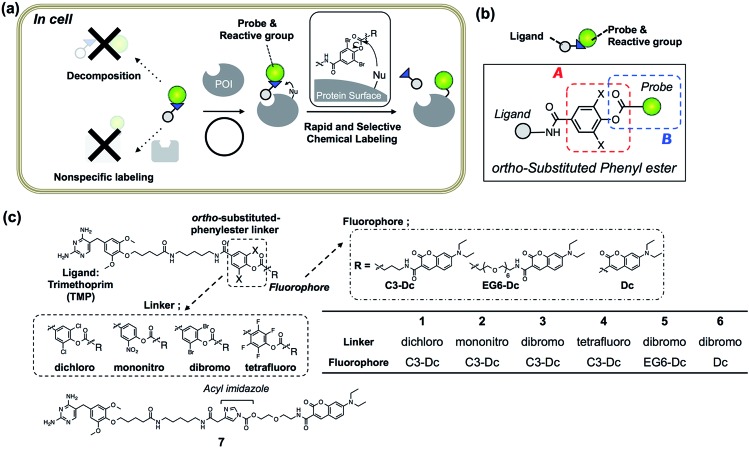
Ligand-directed dibromophenyl benzoate (LDBB) chemistry for rapid and selective intracellular protein labeling. (a) Schematic illustration of LDBB chemistry for chemical labeling of a protein of interest (POI) in living cells. (b) Design strategy for intracellular protein acylation; the reactivity was tuned at position A (*ortho*-substituted phenyl ester moiety), and the stability was tuned at positions A and B (probe moiety). (c) Chemical structures of reagents **1–6** for eDHFR containing TMP ligand and fluorophores, which were linked with various phenyl ester linkers (or acyl imidazole-type reagent **7**).

Toward this goal, we focused on protein acylation using Fenical's LDPE chemistry because the phenyl esters can potentially modify Lys residues, which are often abundant on protein surfaces^8^ and sufficiently reactive under the physiological conditions.^9^ We sought to tune both the reactivity and stability of the LDPE reagents, in order to obtain optimal PE derivatives for “selective but rapid” intracellular protein labeling (Fig. 1a). The reactivity of the substituted PE group was initially controlled by changing the substituent on the phenol ring (Fig. 1b, part A). The stability was optimized by using sterically hindered PE esters composed of an “*ortho*”-substituted phenol and an alkyl/benzoic acid, both of which are expected to be effective for minimizing nonspecific reactions and enzymatic decomposition (Fig. 1b, part B). From the screening of several LDPE reagents, *ortho*-dibromophenyl benzoate derivatives were found to exhibit the moderate reactivity required for selective and rapid protein acylation of dihydrofolate reductase (eDHFR) in *E. coli* cell lysates, as well as *in vitro*. By using this *L*igand-*D*irected di*B*romophenyl *B*enzoate (LDBB) chemistry, we successfully labeled and imaged not only overexpressed eDHFR as a model protein, but also endogenously expressed human carbonic anhydrase II and XII (hCAII, XII) in living mammalian cells.

## Results and discussion

### Molecular design of *ortho*-substituted phenyl ester reagents

*Escherichia coli* dihydrofolate reductase (eDHFR) and trimethoprim (TMP) were chosen as the initial target–ligand pair for screening of the reaction moieties because this ligand–protein binding is known to be specific even in mammalian cells (the *K*_d_ value was about 10 nM), and a reactive Lys is located near the ligand binding pocket of eDHFR.^10–12^ Accordingly, we designed four types of new reagents **1–4** containing a TMP ligand and a synthetic fluorophore (7-diethylaminocoumarin (Dc)), connected with *ortho*-substituted phenyl ester linkers, such as 3,5-dichloro-, 3-nitro-, 3,5-dibromo- and 2,3,5,6-tetrafluoro-4-hydroxy-benzoic acid (Fig. 1c). The theoretical p*K*_a_ values of these leaving groups are 5.72, 5.68, 5.59 and 4.17, and therefore the expected order of the reactivity was **4** > **3** > **2** > **1**. To evaluate the effect of steric hindrance, we also prepared **5**, having a flexible-type ethylene glycol linker between the reactive group and Dc, or **6**, having no linker. Additionally, acyl imidazole-type control compound **7**, having a TMP ligand and Dc fluorophore was prepared.^5^ The compounds were synthesized according to the synthetic protocols shown in the ESI.† Unfortunately, we could not isolate the tetrafluoro-type reagent **4**, because of the low stability of this reactive group under the final condensation conditions.

### Optimization of the reactivity of PE reagents in a test tube

**Fig. 2:**
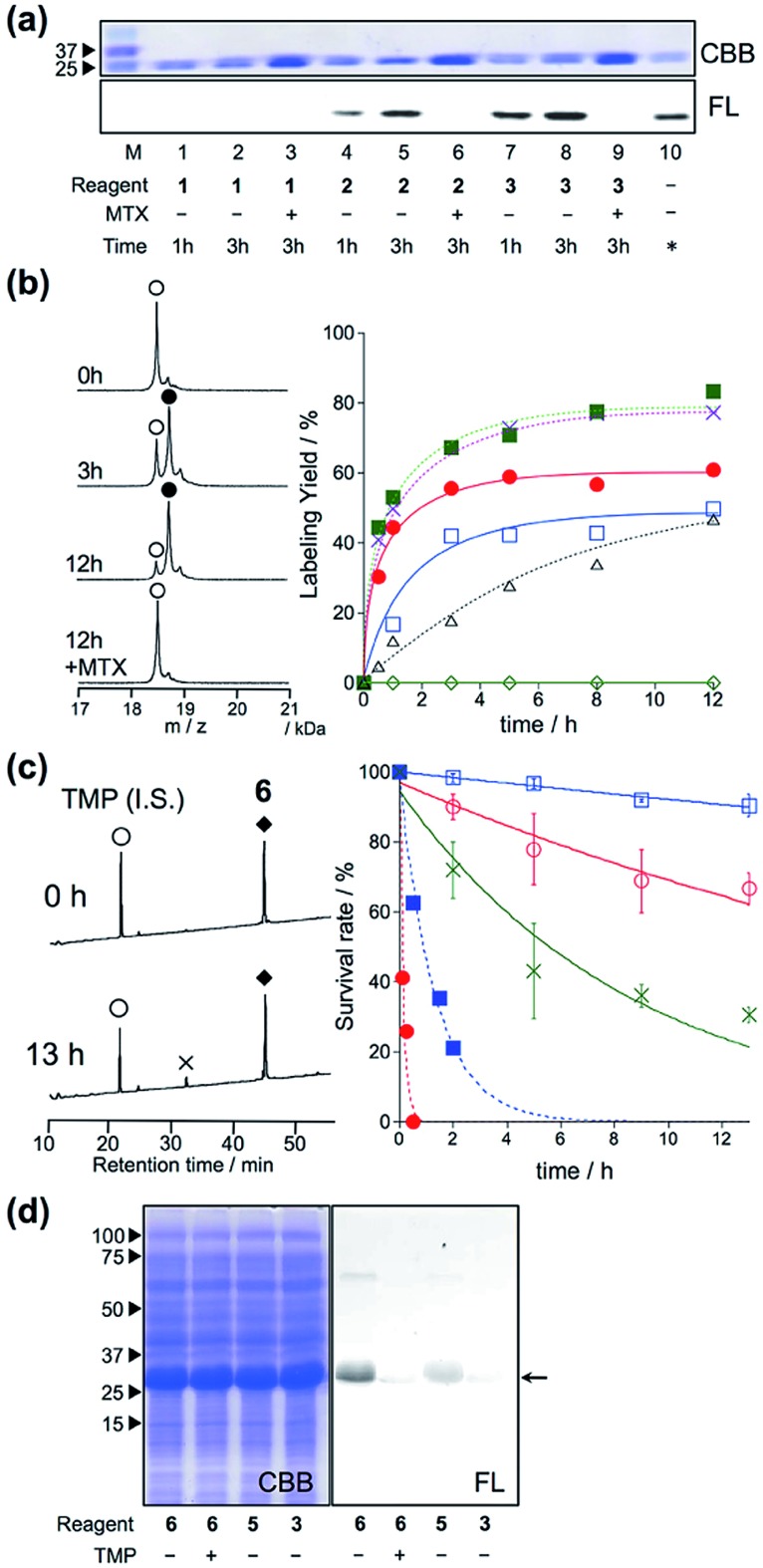
eDHFR-selective labeling *in vitro*. (a) SDS-PAGE analysis of the covalent labeling of purified eDHFR with **1**, **2**, and **3** in a test tube. Reaction conditions: eDHFR (10 μM) and labeling reagents (20 μM) were incubated in the presence or absence of 100 μM MTX in 50 mM HEPES buffer (pH 7.2) at 37 °C. In lane 10, an independently prepared Dc–eDHFR conjugate (60% yield) was used as a standard marker for the determination of the labeling yields. The gel was analyzed by in-gel fluorescence imaging (FL, lower), and stained with Coomassie brilliant blue (CBB, upper). (b) (left) MALDI-TOF MS spectra of eDHFR (10 μM) labeled with compound **6** (20 μM) in 50 mM HEPES buffer (pH 7.2) at 37 °C. 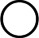 denotes native eDHFR and 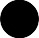 denotes labeled eDHFR. (right) Time profiles of eDHFR (10 μM) labeled with **1** (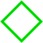), **2** (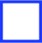), **3** (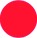), **5** (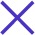), **6** (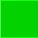) and **7** (LDAI reagent, 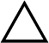) in buffer at 37 °C. The labeling reaction was monitored by MALDI-TOF MS analyses. (c) (left) HPLC charts of labeling reagent **6** (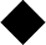) in buffer solution at 37 °C for 0 h (top) or 13 h (bottom) of incubation. 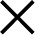 denotes decomposed reagent **6**. 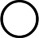 denotes the internal standard. (right) HPLC analyses of the stability of labeling reagents **3**, **5** and **6** in the absence (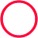; **3**, 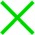; **5**, 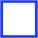; **6**) or presence (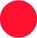; **3**, 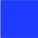; **6**) of porcine liver esterase (100 nM) in buffer solution at 37 °C. (d) SDS-PAGE analysis of the labeling of Trx–His6–eDHFR with **6** (10 μM) in *E. coli* lysates.

*In vitro* experiments were first conducted to examine which is the best reactive group for rapid and selective labeling of eDHFR. The labeling reactions of purified eDHFR (10 μM) with Dc-appended PE reagents **1–3** (20 μM) were analyzed by SDS-PAGE and fluorescence gel imaging. As shown in Fig. 2a, fluorescence was clearly observed from the band for eDHFR after a 3 h incubation with **2** or **3**, whereas no fluorescence was observed upon incubation with **1**. In addition, no significant bands were observed when eDHFR was incubated with **2** or **3** in the presence of methotrexate (MTX, 100 μM), a strong inhibitor of eDHFR, indicating that these labeling reactions were driven by a specific ligand–protein interaction. The detailed labeling kinetics, which were evaluated by MALDI-TOF MS analyses, clearly showed that the initial rate of the labeling reaction with **3** was 3.4-fold faster than that with **2** (Fig. 2b and S1†). These results showed that the *ortho*-dibromophenyl ester (di-Br) type of reactive group was useful for LD chemistry. Next, the spacer effect between Dc and the di-Br-type reactive group was evaluated using **3**, **5** and **6**, which have an alkyl chain, oligoethylene glycol and no spacer, respectively. In all cases, the molecular mass corresponding to Dc-labeled eDHFR was observed as shown in Fig. 2b and S1.† The kinetic analyses of the labeling using these reagents revealed that the initial rates of these reactions were almost the same for all three reagents (0.14, 0.16 and 0.17 μM^–1^ min^–1^ for **3**, **5** and **6**, respectively, Fig. 2b). These were over 7-fold faster than that obtained using acyl imidazole reagent **7** (0.02 μM^–1^ min^–1^ for **7**), indicating that this new LD chemistry is more rapid than LDAI chemistry, at least *in vitro*. We also confirmed that the reaction was site specific using **6** by subjecting the Dc-labeled eDHFR to proteolytic digestion. Conventional peptide mapping analysis revealed that Lys32, located near the ligand-binding site, was predominantly modified (over 94%) by the acylation reaction (Fig. S2†).

### Evaluation of the stability of LDBB reagents *in vitro* and the reaction specificity under crude conditions

Next, we evaluated the stabilities of the reagents under the labeling conditions using HPLC analyses. The half-lives of the reagents in aqueous buffer were 19.5 h and 5.6 h for **3** and **5**, whereas only 10% of **6** was hydrolyzed even after incubation for 13 h at 37 °C (Fig. 2c and S3†). Moreover, upon the addition of an esterase, **3** was completely decomposed after 30 min of incubation, whereas 20% of **6** remained after 2 hours (Fig. 2c and S3†). These results clearly indicated that **6** was resistant toward both autolysis in aqueous buffer, and also catalytic hydrolysis with esterase, maybe because of the steric hindrance provided by the *ortho*-dibromophenyl benzoate (the CPK model is shown in Fig. S4†). Overall, the results clearly demonstrated that ligand-directed dibromophenyl benzoate (LDBB) reagent **6**, which is directly connected to Dc, has moderate reactivity (derived from the leaving-group ability of dibromophenol) and stability (derived from the sterically hindered benzoate structure) for use in a rapid and specific eDHFR labeling reaction *in vitro*. To investigate the labeling selectivity of these reagents under crude conditions, the reactions were carried out using cell lysates of *E. coli* that overexpressed thioredoxin tag fused eDHFR. A clear single fluorescent band corresponding to tag-fused eDHFR appeared when the cells were incubated with **6** (Fig. 2d). On the other hand, no detectable fluorescence was observed upon the addition of an excess amount of TMP, indicating that this labeling reaction was driven by a specific ligand–protein interaction. Using **3** or **5**, the labeling yield was apparently lower than using **6**. Taken together, these results showed that **6** is sufficiently stable and can label eDHFR specifically even in cell lysates containing various reactive biomolecules.

### eDHFR labeling with Dc-type reagents in living mammalian cells

**Fig. 3:**
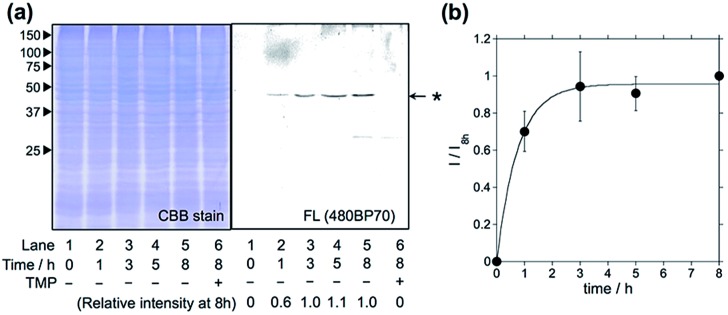
Intracellular eDHFR–GFP labeling with optimized reagent **6** in HeLa-DG cells. (a) SDS-PAGE analyses of the labeling reaction of **6** (1 μM) in HeLa-DG cells. The gel was analyzed by in-gel fluorescence imaging (right) and stained with CBB (left). Lane 6 shows the reaction in the presence of TMP (10 μM). (b) Time course of eDHFR–GFP labeling in HeLa-DG cells. The fluorescence intensity (*I*) was normalized using the intensity for 8 h of incubation (*I*_8h_). The experiments were performed in triplicate to obtain mean and standard deviation values (shown as error bars).

Having optimized the Dc-type of LDBB labeling reagent, we next attempted to modify intracellular eDHFR in mammalian cells. To assess the labeling specificity of the reagents, we used human epithelial HeLa cells stably expressing eDHFR fused with green fluorescent protein (GFP) (HeLa-DG). The HeLa-DG cells were incubated in a medium containing reagent **6** at 37 °C, and washed three times with fresh medium. The cells were then lysed and analyzed by SDS-PAGE, fluorescence gel imaging or western blotting (Fig. 3 and S5†). Notably, a single fluorescence band corresponding to Dc-labeled eDHFR–GFP was detected, despite the presence of various other proteins as shown in the CBB staining image. The addition of TMP ligand completely blocked the labeling, which indicates that this modification occurs *via* a specific ligand–eDHFR interaction even inside cells. The labeling yield was determined to be 85 ± 5% of the total eDHFR–GFP, which was calculated from the fluorescence intensity of Dc and the chemiluminescence intensity of anti-GFP antibody (Fig. S5c†). The time-dependence experiments revealed that the new reagent **6** labeled 50% of eDHFR–GFP with a *t*_1/2_ of 30 min (Fig. 3b). Conversely, several proteins other than eDHFR–GFP were also labeled nonspecifically by using **5**, whereas no eDHFR labeling was observed by using **3** (Fig. S6a†). These results may be explained by the lower selectivity or reactivity of reagents **3** and **5** compared with **6**, similar to results obtained for the *in vitro* experiments. In addition, LDAI reagent **7** did not label intracellular eDHFR–GFP (Fig. S6b†), perhaps because of the lower stability or cellular permeability of this reagent. Together, these results clearly demonstrated that the rapid and quantitative labeling of an intracellular natural protein could be achieved using LDBB chemistry in living mammalian cells.

### Live-cell imaging of native eDHFR by covalent modification of a synthetic fluorophore

**Fig. 4:**
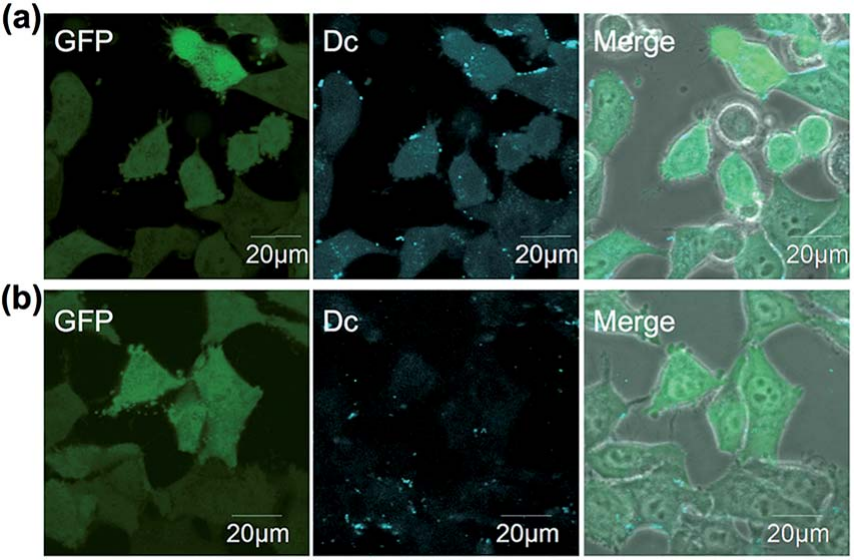
(a and b) Live-cell imaging of eDHFR–GFP with **6** (50 nM) in the absence (a) or presence (b) of TMP (50 μM) in HeLa-DG cells. Fluorescence images of GFP, Dc and their merged phase contrast images are displayed in the left, middle and right of each panel, respectively. Images of Dc were obtained at an excitation of 405 nm using a 420–480 nm emission filter, those of GFP were obtained at an excitation of 473 nm using a 490–520 nm emission filter. Scale bar: 20 μm.

The in-cell eDHFR–GFP labeling results led us to investigate whether LDBB chemistry could be applied to intracellular protein imaging by covalent modification of a synthetic fluorophore. HeLa-DG cells were incubated in a medium containing reagent **6** (50 nM) for 3 h at 37 °C, then washed three times with fresh medium, and the cells were observed by confocal laser scanning microscopy (CLSM). From the CLSM images of the HeLa-DG cells using the GFP channel, eDHFR–GFP was found to be distributed throughout the intracellular region, including the cytosol and nuclei. As shown in Fig. 4a, the fluorescence of Dc was clearly detected inside the cells and merged with the GFP image. However, before the labeling reaction, the addition of TMP resulted in the disappearance of Dc fluorescence (Fig. 4b). These results clearly show that the new LDBB reagent **6** could be used to specifically label and visualize intracellular eDHFR–GFP using a synthetic fluorophore.

**Fig. 5:**
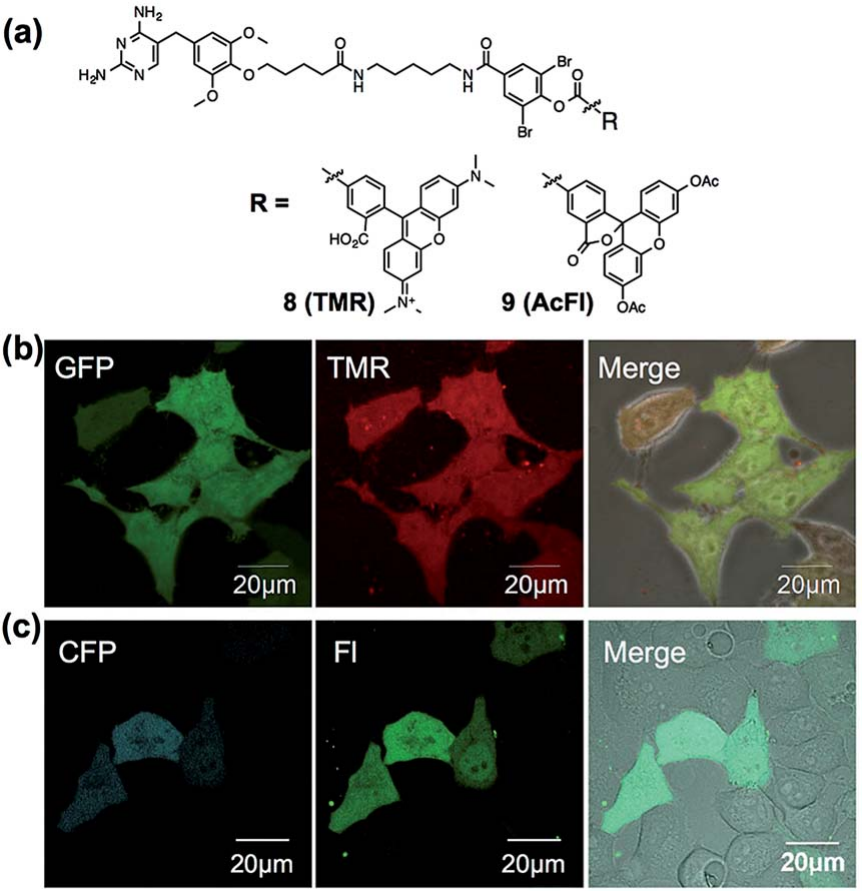
(a) Chemical structures of LDBB reagents **8** and **9**. (b) Live-cell imaging of eDHFR–GFP with **8** (20 nM) in HeLa-DG cells. Fluorescence images of GFP, TMR and their merged phase contrast images are displayed in the left, middle and right of each panel, respectively. Images of GFP were obtained at an excitation of 473 nm using a 490–520 nm emission filter and those of TMR were obtained at an excitation of 559 nm using a 570–670 nm emission filter, respectively. (c) Live-cell imaging of eDHFR-CFP by **9** (50 nM) in HeLa cells expressing eDHFR-CFP. Fluorescence images of CFP, fluorescein (FL) and their merged differential interference contrast images are displayed in the left, middle and right of each panel, respectively. Images of CFP were obtained at an excitation of 457 nm using a 470–490 nm emission filter and those of fluorescein (FL) were obtained at an excitation of 515 nm using a 520–620 nm emission filter. Scale bar: 20 μm.

To assess the applicability of fluorophores other than Dc in LDBB chemistry, we prepared two more fluorophore-appended reagents for the labeling of intracellular eDHFR (Fig. 5a, tetramethylrhodamine (TMR, **8**) and acetylfluorescein (AcFl, **9**)), according to the molecular design principle of LDBB chemistry as discussed above.^13,14^ Fig. 5b shows that the TMR-type of reagent **8** was useful to visualize the localization of eDHFR–GFP in live HeLa-DG cells to a similar degree as **6**. Reagent **9** can also label and image cyan fluorescent protein-fused eDHFR (eDHFR-CFP), which was transiently expressed in live HeLa cells (Fig. 5c) (we chose CFP as an alternative marker to GFP because the emissions of fluorescein and GFP overlap). These results clearly show that LDBB chemistry is suitable for fluorescent live-cell imaging of intracellular natural protein with blue, green and red emission, and this imaging tool may be applicable for concomitant use with various other imaging technologies, such as fluorescent protein tags or organelle staining probes.

### Endogenous intracellular protein labeling and imaging using a LDBB reagent

**Fig. 6:**
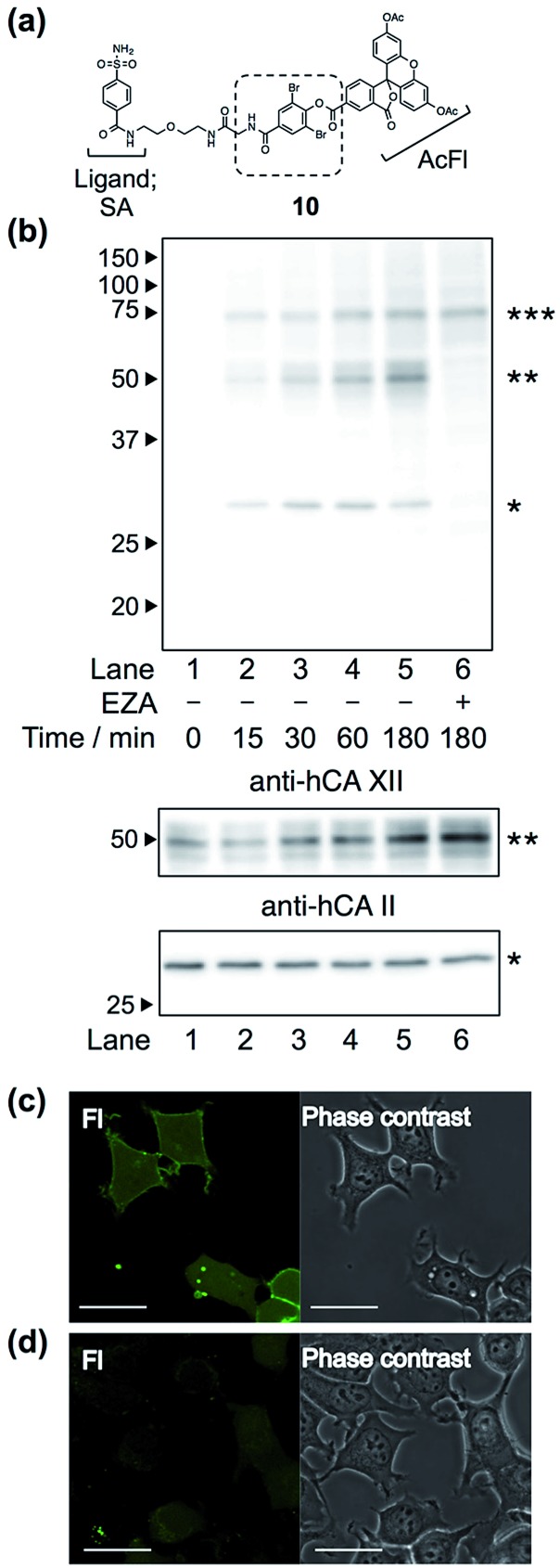
(a) Chemical structure of reagent **10**, having a benzenesulfonamide (SA) ligand for hCA labeling. (b) Western blotting analyses of the labeling of endogenous hCA with **10** (100 nM) in living MCF7 cells, detected with anti-fluorescein antibody (top), anti-hCAXII antibody (middle) and anti-hCAII antibody (bottom). The band with a single asterisk (*) corresponds to hCAII, the double asterisk (**) corresponds to hCAXII and the triple asterisk (***) corresponds to a non-specifically-labeled BSA derived from the cultured medium. (c and d) Live-cell imaging of hCA labeling with **10** (100 nM) in MCF7 cells in the absence (c) or presence (d) of EZA (100 μM). Fluorescence images and phase contrast images of fluorescein (FL) are displayed in the left and right of each panel, respectively. Scale bar: 30 μm.

Finally, we attempted to label “endogenous” protein in live cells by altering the affinity ligand, owing to the modular design of LDBB reagents. Human carbonic anhydrase II (hCAII), a cytosolic protein, and XII (hCAXII), a membrane-bound protein, were chosen as the target proteins endogenously expressed in MCF7 cells (breast cancer cell line). Accordingly, we synthesized the new AcFl-type LDBB reagent **10** containing benzenesulfonamide as the selective ligand for the hCA family (Fig. 6a, *K*_i_ of 260 nM).^15,16^ MCF7 cells were incubated with **10** at 37 °C, and then the labeling of hCA was evaluated by western blotting analyses. Fig. 6b shows that after only 15 to 30 min of incubation, two distinct bands could be detected at ∼30 kDa and ∼50 kDa using anti-fluorescein antibody, corresponding to the band obtained from western blotting with the anti-hCAII antibody (∼30 kDa) and anti-hCAXII antibody (∼50 kDa). No band was observed in the presence of ethoxzolamide (EZA, a strong inhibitor of the hCA family of enzymes), which clearly demonstrated that **10** can covalently label not only intracellular hCAII but also cell-surface hCAXII with strict specificity in live cells.^17^ Using the SA-type LDBB reagent *in vitro*, the labeling sites for hCAI or II were identified as Lys171 or Lys169/His3, respectively (Fig. S9 and S10†). These amino acids were again located near the binding site of each target protein (Fig. S9e and S10e†), demonstrating that the LDBB reagents were able to react not only with lysine, but also with (at least) histidine, based on the proximity effect. These results also implied the general applicability of the LDBB chemistry for the efficient labeling of various proteins having nucleophilic amino acids appropriately located near the ligand binding site. The CLSM images also showed that strong fluorescence was clearly observed from both the cell membrane and cytosolic part of MCF7 cells. In contrast, no fluorescence was detected in the presence of EZA. Taken together, these results clearly demonstrate that the LDBB chemistry is a sufficiently rapid, efficient and specific labeling technique for endogenous protein imaging both on the cell surface and inside the cell.

## Conclusion

Herein, we demonstrated that LDBB chemistry is useful for the selective and rapid labeling of not only overexpressed proteins, but also endogenous proteins in mammalian cells. By finely tuning the reactivity and stability of the phenyl ester moiety, it was found that *ortho*-dibromophenyl benzoate was sufficiently stable in aqueous buffer (decomposed less than 10% after 13 h incubation), yet highly reactive in living cells and *in vitro* (over 85% labeling yield was achieved for overexpressed eDHFR in mammalian cells). Moreover, owing to the reactive amino acid preferences and the modular design principle, the LDBB reagents can label various natural (or engineered) proteins having nucleophilic amino acids (including at least lysine and histidine) located near the ligand binding site, as shown in the cases of the labeling of eDHFR and hCAs.^11*c*^ The present study also showed that LDBB chemistry is a powerful tool for natural protein imaging in living mammalian cells, because of the fact that various benzoate-type fluorophores (including Dc, FL and TMR) can be easily installed on the target proteins with sufficient selectivity and rapidity. We envision that the extension of this ligand-directed chemistry should allow for multi-color imaging of various endogenous proteins and analyses of protein–protein interactions in living cells in the future.

## Experimental procedures

### Synthesis

All synthetic procedures and compound characterization are described in the ESI.†

### General materials and methods

All chemical reagents and solvents were obtained from commercial suppliers (Aldrich, Tokyo Chemical Industry (TCI), Wako Pure Chemical Industries, Acros Organics, Sasaki Chemical, or Watanabe Chemical Industries) and used without further purification. UV-visible spectra were recorded on a Shimadzu UV-2550 spectrometer. All reactions were carried out under an atmosphere of argon or nitrogen unless otherwise noted. Thin layer chromatography (TLC) was performed on silica gel 60 F254 precoated aluminum sheets (Merck) and visualized by fluorescence quenching or ninhydrin staining. Chromatographic purification was accomplished using flash column chromatography on silica gel 60 N (neutral, 40–50 mm, Kanto Chemical). ^1^H NMR spectra of samples were recorded on Varian Mercury 400 (400 MHz) spectrometers. ^13^C NMR spectra of samples were recorded on Jeol ECX-400P (100 MHz) spectrometers. IR spectra were recorded on a JASCO FT/IR-410. Matrix-assisted laser desorption/ionization time-of-flight mass spectrometry (MALDI-TOF MS) spectra were recorded on an Autoflex III instrument (Bruker Daltonics) using α-cyano-4-hydroxycinnamic acid (CHCA) as the matrix. High-resolution mass spectra were measured on an Exactive (Thermo Scientific, CA, USA) equipped with electron spray ionization (ESI). SDS-PAGE and western blotting were performed using a Bio-Rad Mini-Protean III electrophoresis apparatus. Fluorescence and chemical luminescent signals were detected with Imagequant LAS 4000 (GE Healthcare).

### *In vitro* labeling of eDHFR

*E. coli* DHFR (eDHFR) was prepared and purified as previously reported.^18^ Briefly, thioredoxin- and His-tag fused eDHFR (Trx–His6–eDHFR) was purified with TALON metal affinity resin, and the purified proteins were subjected to site-specific cleavage with thrombin, and then purified with TALON metal affinity resin and benzamidine sepharose to remove Trx-His6 and thrombin. The concentration of eDHFR was determined by absorbance at 280 nm using a molar extinction coefficient of 31 100 M^–1^ cm^–1^. The solution of eDHFR (10 μM) was incubated with the labeling reagent (20 μM) in the absence or presence of MTX (100 μM) in 50 mM HEPES buffer (pH 7.2) and incubated at 37 °C. Aliquots were taken at different time points, mixed with MTX (100 μM), and subjected to SDS-PAGE analyses. Fluorescence gel images were acquired using a Bio-Rad ChemiDoc XRS system with a 480BP80 filter, and analyzed with Quantity One 1-D Analysis Software (Bio-Rad Laboratories). In separate experiments using MALDI-TOF MS analyses (matrix, sinapinic acid), the labeling yield was estimated by determining the relative MS peak intensity of labeled eDHFR to unreacted eDHFR. Aliquots were taken at different time points, quenched by using ZipTip purification with 0.1% TFA aq., and spotted onto a MALDI plate with the matrix solution.

### In cell labeling of eDHFR

Before labeling, HeLa-DG cells (6 × 10^5^ cells) were seeded in a 35 mm dish, incubated in DMEM (10% fetal bovine serum (FBS)) for 24 h at 37 °C, and then washed three times with phosphate buffered saline (PBS). The cells were treated with the LDBB reagent (100 or 500 nM) at 37 °C in DMEM (FBS free, 1 mL) and incubated. As a control experiment, the labeling was conducted in the presence of TMP (10 μM, pre-incubated for 5 min). After labeling, the cells were washed three times with PBS, and then RIPA buffer (pH 7.4, 25 mM Tris–HCl, 150 mM NaCl, 0.1% SDS, 1% Nonidet P-40, 0.25% deoxycholic acid) was added containing 1% protease inhibitor cocktail set III (Calbiochem®) and 10 μM MTX. The lysed sample was collected and centrifuged (10 000 *g*, 10 min at 4 °C). The supernatant was mixed with the same volume of 2× sampling buffer (pH 6.8, 125 mM Tris–HCl, 20% glycerol, 4% SDS, 0.01% bromophenol blue, 8% 2-mercaptoethanol) and incubated for 5 min at 95 °C. The samples were subjected to SDS-PAGE (12.5%) and in-gel fluorescence images were obtained. In the live cell imaging experiments, the cells (2 × 10^5^ cells, pre-cultured for 24 h in a 35 mm glass bottomed dish) were washed three times with PBS, and then treated with LDBB reagent **6** (50 nM) at 37 °C in DMEM (FBS free, 1 mL) and incubated for 8 h. After removing the medium, the cells were treated with DMEM (10% FBS) for 3 h, and then subjected to imaging analysis using a CLSM (Olympus, FLUOVIEW FV10i). Images of Dc were obtained at an excitation of 405 nm using a 420–480 nm emission filter, and those of GFP were obtained at an excitation of 473 nm using a 490–520 nm emission filter. For the TMR-type reagent **8**, the HeLa-DG cells were labeled with **8** (20 nM) at 37 °C in DMEM (FBS free, 1 mL) and incubated for 3 h. After removing the medium, the cells were treated with DMEM (10% FBS) for 5 h, and then subjected to imaging analysis using a CLSM (FV10i). Images of GFP were obtained at an excitation of 473 nm using a 490–520 nm emission filter and those of TMR were obtained at an excitation of 558 nm using a 570–670 nm emission filter, respectively. For the AcFl-type reagent **9**, the normal HeLa cells (2 × 10^5^ cells) were cultured in DMEM (10% FBS) in a 35 mm glass-bottomed dish at 37 °C for 24 h. The cells were transiently transfected with the peDHFR-ECFP plasmid using a FuGENE HD (Promega). After 24 h of transfection, the cells were labeled with **9** (50 nM) at 37 °C in DMEM (FBS free, 1 mL) and incubated for 3 h. After removing the medium, the cells were treated with DMEM (10% FBS) for 5 h, and then subjected to imaging analysis using a CLSM (Olympus, FLUOVIEW FV1000). Images of CFP were obtained at an excitation of 457 nm using a 470–490 nm emission filter, and those of fluorescein (FL) were obtained at an excitation of 515 nm using a 520–620 nm emission filter.

### *In vitro* labeling of hCAI and II

Human carbonic anhydrase I and II (hCAI, II) were purchased from SIGMA-Aldrich, and used without further purification. The concentrations of hCAI and II were determined by absorbance at 280 nm using a molar extinction coefficient of 49 000 M^–1^ cm^–1^ for hCAI and 54 000 M^–1^ cm^–1^ for hCAII in 50 mM HEPES buffer (pH 7.4, 100 mM NaCl). The solution of hCAI or II (20 μM) was incubated with labeling reagent **11** (40 μM) at 37 °C. After 10 h, the labeling reaction was confirmed with MALDI-TOF MS analyses (matrix: sinapinic acid), and the labeling yield was determined to be 46% for hCAI and 38% for hCAII. After labeling, the protein was purified with gel filtration (TOYO PEARL). To this solution was added urea (at a final concentration of 2 M) and lysyl endopeptidase (LEP) (LEP/substrate ratio = 1/30 (w/w)). After incubation at 37 °C overnight, the digested samples were used for RP-HPLC. The collected fractions were analysed by MALDI-TOF MS using CHCA as the matrix and the labeled fragment was further characterized by MALDI TOF-TOF MS/MS (Bruker Daltonics) or MALDI-LTQ-Orbitrap-MS/MS (Thermo Scientific, CA, USA).

### In cell labeling of hCAII and XII

Before labeling, MCF7 cells (2 × 10^5^ cells) were seeded in a 35 mm dish, incubated in DMEM (10% fetal bovine serum (FBS)) for 24 h at 37 °C, and then washed three times with DMEM (FBS free). The cells were treated with LDBB reagent **10** (100 nM) at 37 °C in DMEM (FBS free, 1 mL) and incubated. As a control experiment, the labeling was conducted in the presence of EZA (100 μM, pre-incubated for 5 min). After labeling, the cells were washed three times with PBS, and then RIPA buffer was added containing 1% protease inhibitor cocktail set III (Calbiochem) and 100 μM EZA. The lysed sample was collected and centrifuged (10 000 *g*, 10 min at 4 °C). The supernatant was mixed with the same volume of 2× sampling buffer (pH 6.8, 125 mM Tris–HCl, 20% glycerol, 4% SDS, 0.01% bromophenol blue, 100 mM DTT) and vortexed for 1 h at room temperature. The samples were resolved by 12.5% SDS-PAGE and electrotransferred onto an Immun-Blot PVDF membrane (Bio-Rad). The labeled products were detected with anti-fluorescein antibody (Abcam, ×3000) and anti-rabbit IgG antibody–HRP conjugate (GE Healthcare, ×5000). The immunodetection of hCAII used an anti-hCAII antibody (Abcam, ×2000) and anti-rabbit IgG antibody–HRP conjugate (GE Healthcare, ×5000). The immunodetection of hCAXII used an anti-hCAXII antibody (Cellsignaling, ×2000) and anti-rabbit IgG antibody–HRP conjugate (GE Healthcare, ×5000). The HRP signal was detected with a LAS 4000 imaging system (FujiFilm) using ECL plus western blotting detection reagents (GE Healthcare). In the live cell imaging experiments, the MCF7 cells (2 × 10^5^ cells, pre-cultured for 24 h in a 35 mm glass-bottomed dish) were washed three times with DMEM (FBS free), and then treated with LDBB reagent **10** (100 nM) at 37 °C in a DMEM (FBS free, 1 mL) and incubated for 1 h. After removing the medium, the cells were washed three times with DMEM (10% FBS), and then subjected to imaging analysis using a CLSM (Olympus, FLUOVIEW FV10i).
