# Supplementary material for: Ligand-directed dibromophenyl benzoate chemistry for rapid and selective acylation of intracellular natural proteins
Source: Chem Sci. 2015 Mar 23;6(5):3217–24. doi: 10.1039/c5sc00190k (PMC5490417; doi:10.1039/c5sc00190k)
Supplement: Supplementary file 1 [file SC-006-C5SC00190K-s001.pdf]

Electronic Supplementary Material (ESI) for Chemical Science.
This journal is © The Royal Society of Chemistry 2015

Supplementary Information:

Ligand-directed Dibromophenyl Benzoate Chemistry for Rapid and Selective Acylation of Intracellular Natural Proteins

Yousuke Takaoka,[a,b] Yuki Nishikawa,[a] Yuki Hashimoto,[a] Kenta Sasaki,[a] Itaru Hamachi[*a,c]*

[a]*Department of Synthetic Chemistry and Biological Chemistry, Graduate School of Engineering Kyoto University, Katsura, Kyoto 615-8510, Japan.*

[b]*Present address: Department of Chemistry, Graduate School of Science, Tohoku University, Aramaki-aza Aoba 6-3, Aoba-ku, Sendai 980-8578, Japan.*

[c]*Core Research for Evolutional Science and Technology (CREST), Japan Science and Technology Agency, 5 Sanbancho, Chiyoda-ku, Tokyo 102-0075, Japan.*

*Correspondence should be addressed to I.H (ihamachi@sbchem.kyoto-u.ac.jp)

Supplementary Figures

Figure S1. MALDI-TOF MS analyses of eDHFR labeling with **1**, **2**, **3**, **5** and **6**.

Figure S2. Labeling site of eDHFR by the reaction with LDBB reagent **6**.

Figure S3. HPLC analyses of the stabilities of LDBB reagents.

Figure S4. CPK models of reagents **3** and **6**.

Figure S5. eDHFR-GFP labeling with optimized reagent **6** in HeLa-DG cells.

Figure S6. SDS-PAGE analyses of the labeling of eDHFR-GFP with **6**, **5**, **3** or **7** in living HeLa-DG cells.

Figure S7. eDHFR labeling with reagent **8** or **9-2** in test tube.

Figure S8. SDS-PAGE analyses of the labeling of eDHFR-GFP with **8** or **9** in HeLa cells.

Figure S9. Labeling site of hCAI by the reaction with LDBB reagent **11**.

Figure S10. Labeling site of hCAII by the reaction with LDBB reagent **11**.

Synthesis of reagents

References

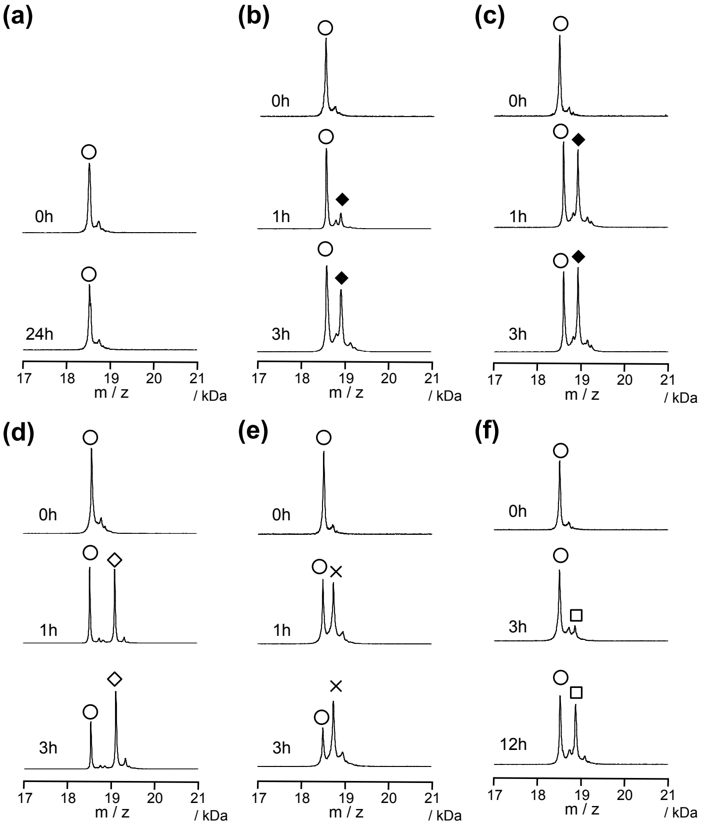

Figure S1. MALDI-TOF mass spectra of a reaction mixture containing purified eDHFR (10 μM) and each reagent (20 μM, **1** (a), **2** (b), **3** (c), **5** (d), **6** (e) or **7** (f)) in 50 mM HEPES buffer (pH 7.2) at 37°C. (○) Native eDHFR (Mw = 18,659); (◆) C3-Dc-labeled eDHFR (Mw = 18,987); (◇) EG6-Dc-labeled eDHFR (Mw = 19,237); (×) Dc-labeled eDHFR (Mw = 18,902); (□) EG1-Dc-labeled eDHFR (Mw = 19,035).

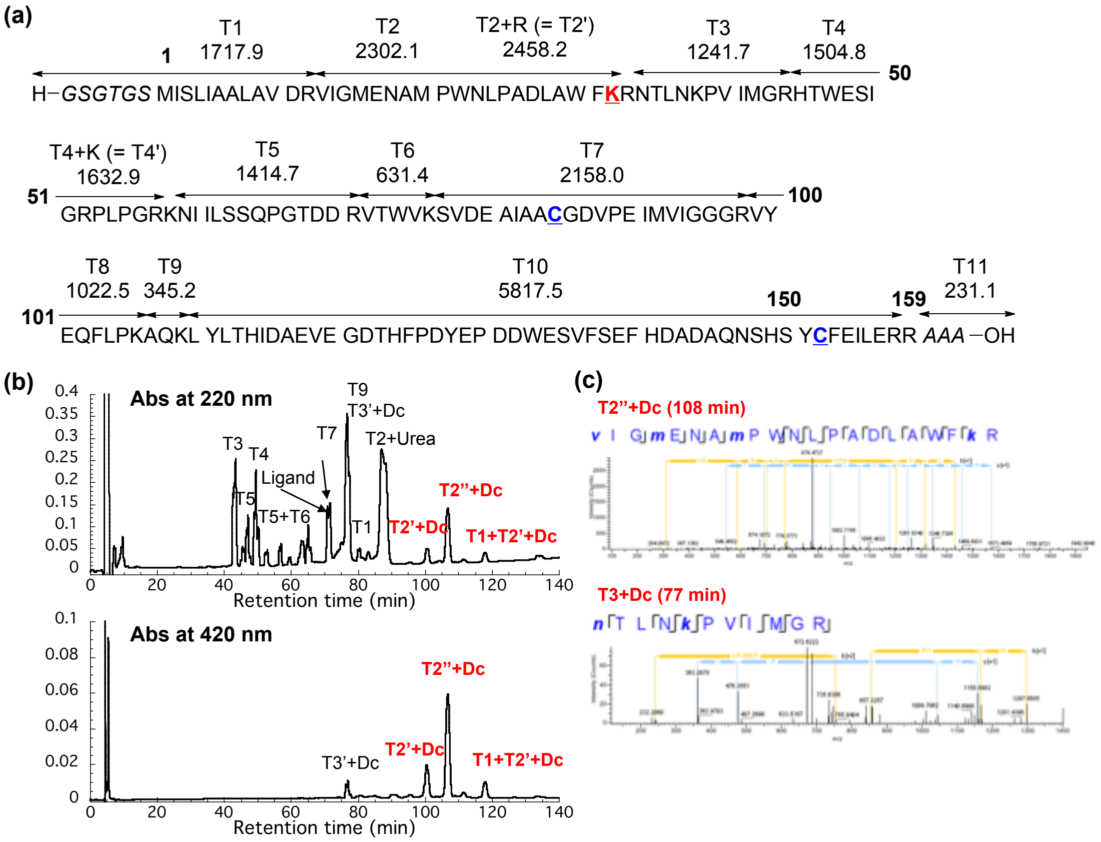

Figure S2. Mass spectral analysis of the labeling site of eDHFR by the reaction with LDBB reagent **6**. (**a**) The primary sequence of eDHFR and the predictable fragments generated by tryptic digestion. The amino acids labeled with **6** are shown in red. (**b**) HPLC analysis of the digested fragments of eDHFR. The chromatograms were shown for the fragments derived from the **6**-labeled eDHFR detected with UV absorption at 220 nm (top) or 420 nm (bottom). The peaks marked with the character (e.g. T1) correspond to the labeled fragments, which were characterized by MALDI-TOF MS analysis. MALDI-TOF MS (CHCA) of peak T3'+Dc (77 min, carbamoylated at N-terminus): calcd. for $[M+2H]^{2+}$ = 764.9007, obsd. 764.8995, peak T2'+Dc (101 min, carbamoylated at N-terminus and oxidized at single Met): calcd. for $[M+2H]^{2+}$ = 1373.1665, obsd. 1373.1687, peak T2''+Dc (108 min, carbamoylated at N-terminus and oxidized at double Met): calcd. for $[M+3H]^{3+}$ = 926.4458, obsd. 926.4453, T1+T2'+Dc (118 min, carbamoylated at N-terminus): calcd. for $[M+H]^{+}$ = 4462.2453, obsd. 4462.2448. (**c**) MALDI-Orbitrap MS/MS analysis of the dye-labeled T2''+Dc (top, 108 min in (b)) or T3'+Dc (bottom, 77 min in (b)).

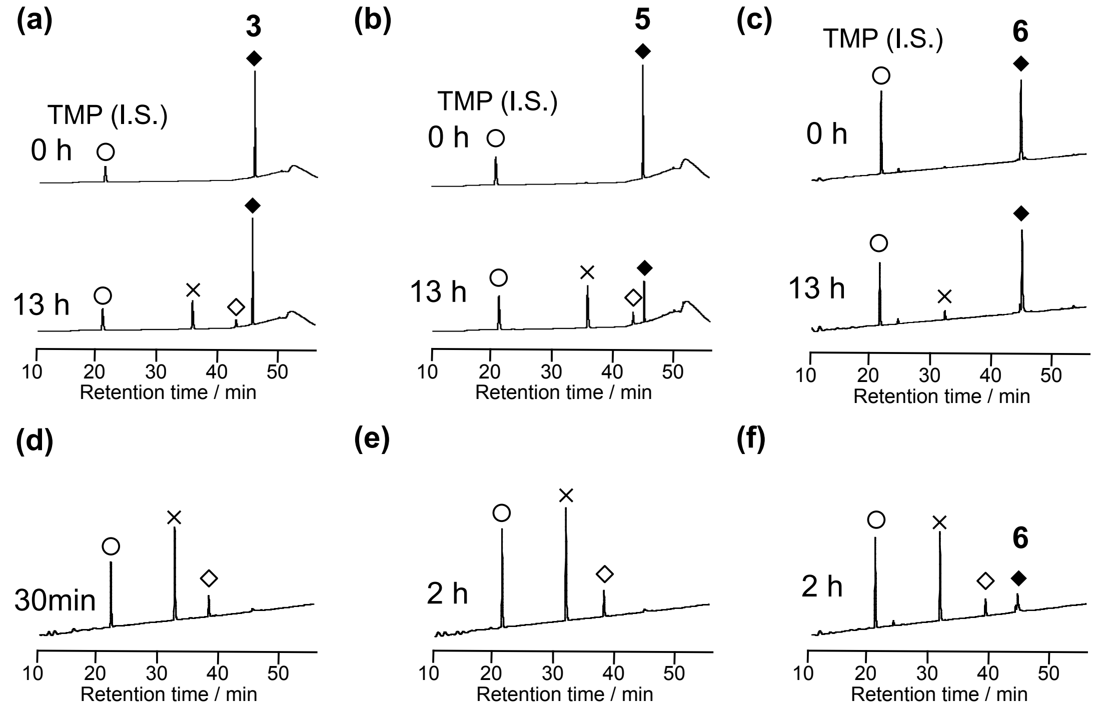

Figure S3. HPLC analyses of the stabilities of LDBB reagents. (a-c) HPLC charts of reagent **3** (a), **5** (b) or **6** (c) incubated in 50 mM HEPES buffer (pH 7.2) at 37ºC. (d-f) HPLC charts of reagent **3** (d), **5** (e) or **6** (f) incubated with porcine liver esterase (100 nM) in 50 mM HEPES buffer (pH 7.2) at 37ºC. Gradients; A (H_2O containing 0.1% TFA) : B (CH_3CN containing 0.1% TFA) = 0 : 100 (0 min) to 10 : 90 (10 min) to 75 : 25 (60 min). (O) internal standard (trimethoprim (TMP), 22 min); (◆) each reagent (about 46 min); (◇) and (x) decomposed reagent (32 or 38 min).

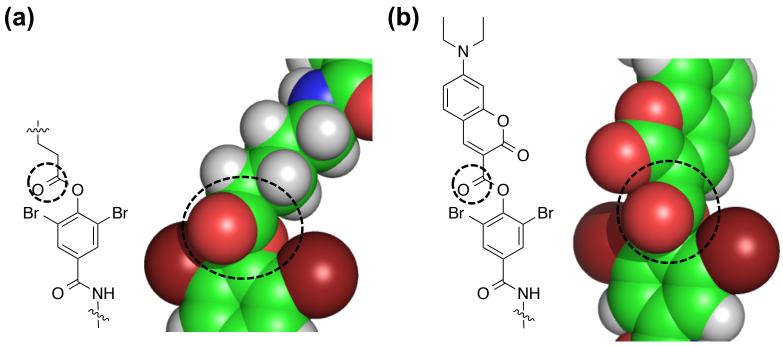

Figure S4. CPK models of reagents **3** (a) and **6** (b). The reactive carbonyl carbons were denoted in dotted circle. Clearly, the reactive group of **6** was sterically hindered rather than **3**.

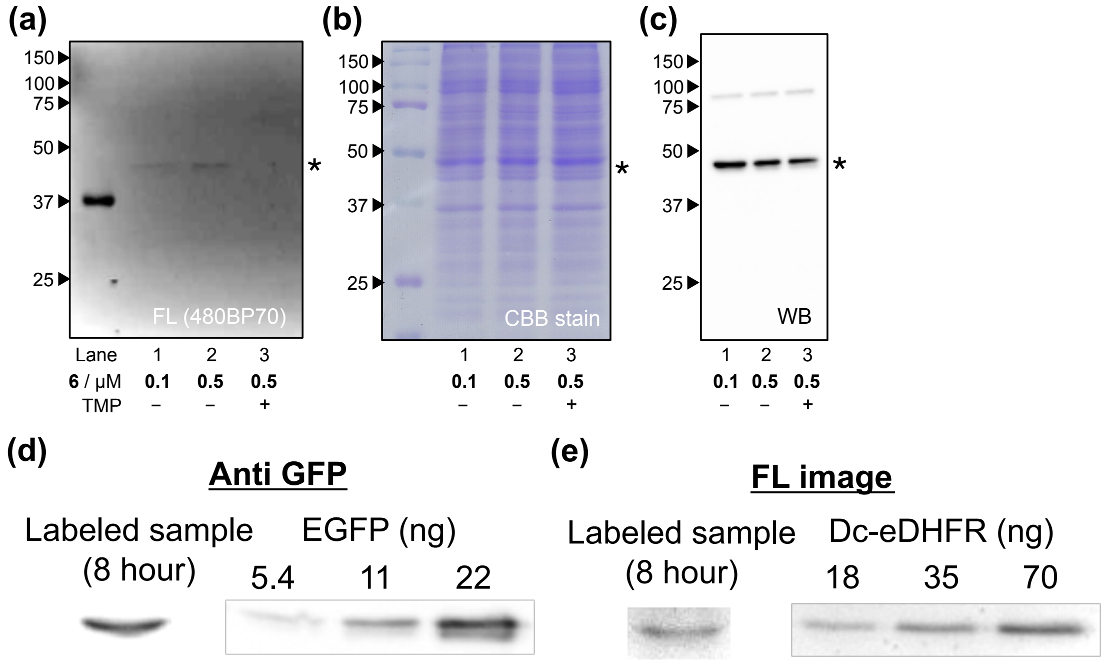

Figure S5. (a-c) SDS-PAGE analysis of the labeling of eDHFR-GFP with **6** (0.1 or 0.5 μM) for 8 h incubation at 37ºC in living HeLa-DG cells. (a, b) The gel was analyzed by in-gel fluorescence imaging (a, FL) and stained with Coomassie brilliant blue (b, CBB). (c) Western blotting analysis of eDHFR-GFP by using anti-GFP antibody in HeLa-DG cells. The band of astarisk (*) was eDHFR-GFP. (d, e) calibration of the labeling yield of **6**-labeled eDHFR-GFP in cells. The quantity of eDHFR-GFP in HeLa-DG cells were calculated from GFP as an internal standard for Western blotting analyses (d) and the quantity of diethylaminocoumarin labeled on eDHFR-GFP in HeLa-DG cells (e) (8h incubation with **6**, the same condition as figure 3a) was calculated from the fluorescence intensity of Dc-labeled eDHFR (prepared in test tube settings with **6** and eDHFR). These experiments were performed triplicate and the mean labeling yield was determined to be 85 ± 5%.

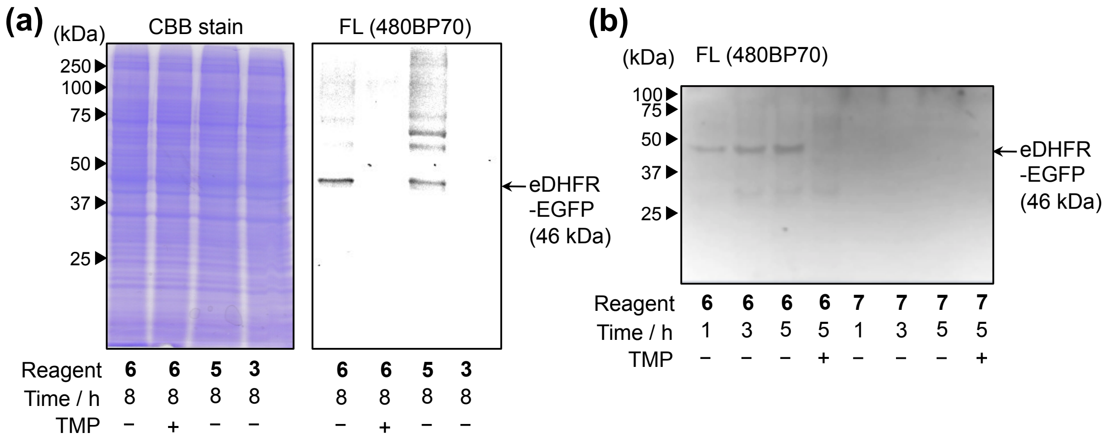

Figure S6. (a) SDS-PAGE analyses of the labeling of eDHFR-GFP with **6**, **5** or **3** (1 μM) in living HeLa-DG cells. The gel was analyzed by in-gel fluorescence imaging (right, FL) and stained with Coomassie brilliant blue (left, CBB). (b) In-gel fluorescence image of the labeling of eDHFR-GFP with **6** or **7** (1 μM) in living HeLa-DG cells.

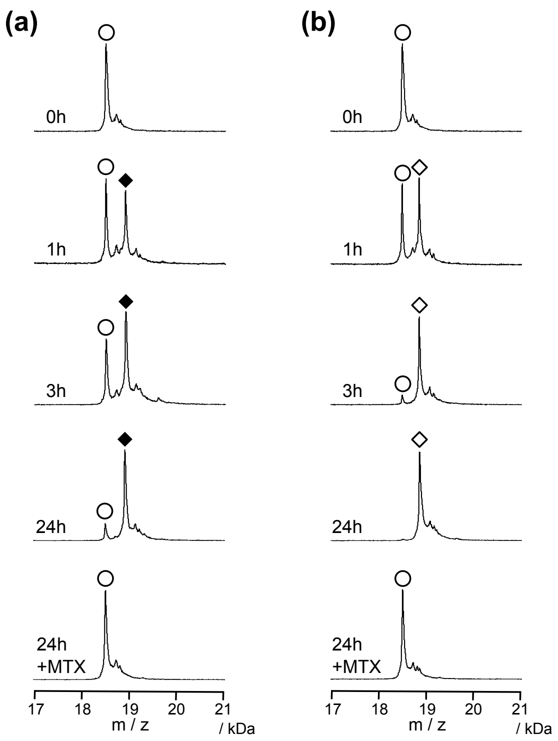

Figure S7. (a, b) MALDI-TOF mass spectra of a reaction mixture containing purified eDHFR (10 μM) and each reagent (20 μM, **8** (a) or **9-2** (b)) in 50 mM HEPES buffer (pH 7.2) at 37ºC. (○) Native eDHFR (Mw = 18,659); (◆) TMR-labeled eDHFR (Mw = 19,072); (◇) Fl-labeled eDHFR (Mw = 19,018). (c) Time profiles of eDHFR (10 μM) labeling with **8** (●, red) or **9-2** (■, blue) in buffer at 37ºC. The labeling reaction was monitored by MALDI-TOF MS analyses (as shown in (a) and (b)).

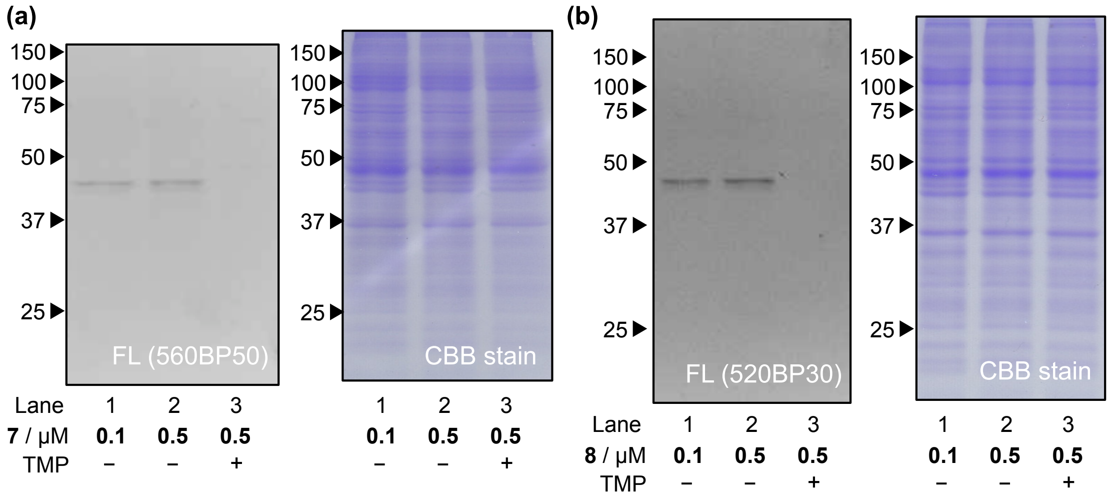

Figure S8. SDS-PAGE analyses of the labeling of eDHFR-GFP with **8** (a) (0.1 or 0.5 μM) or with **9** (b) (0.1 or 0.5 μM) in living HeLa-DG cells in the absence or presence of TMP (10 μM). The gel was analyzed by in-gel fluorescence imaging (FL, left) and stained with Coomassie brilliant blue (CBB, right).

.

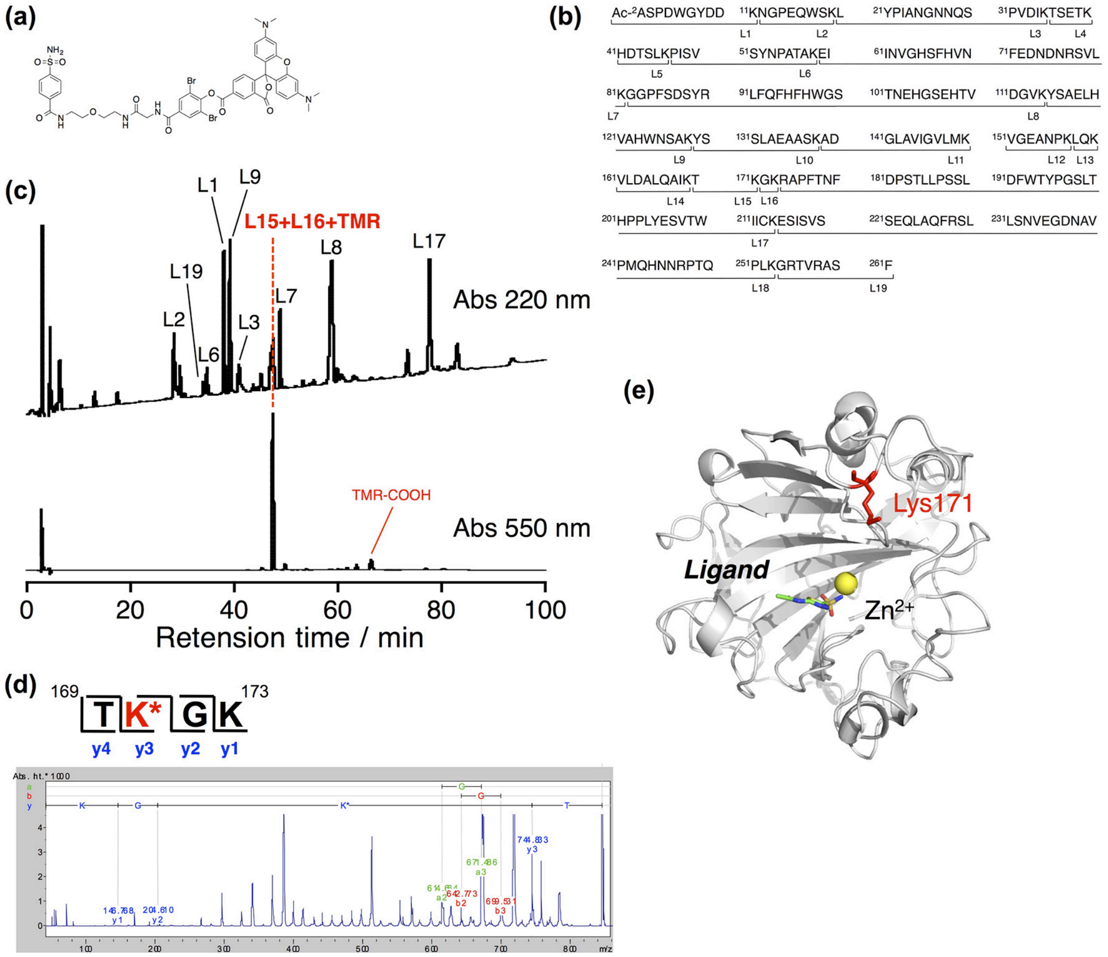

Figure S9. Mass spectral analysis of the labeling site of hCAI by the reaction with LDBB reagent **11**. **(a)** Chemical structure of LDBB reagent **11**. (**b**) The primary sequence of hCAI and the predictable fragments generated by LysC digestion. (**c**) HPLC analysis of the digested fragments of hCAI. The chromatograms were shown for the fragments derived from the **11**-labeled hCAI detected with UV absorption at 220 nm (top) or 550 nm (bottom). The peaks marked with the character (e.g. L1) correspond to the labeled fragments, which were characterized by MALDI-TOF MS analysis. (**d**) MALDI-TOF MS/MS analysis of the dye-labeled L15+L16+TMR. (**e**) 3D structure of hCAI (the labeling site (Lys171) was shown in red, Zn ion was in yellow, and the ligand in green stick, PDB ID: 1AZM).

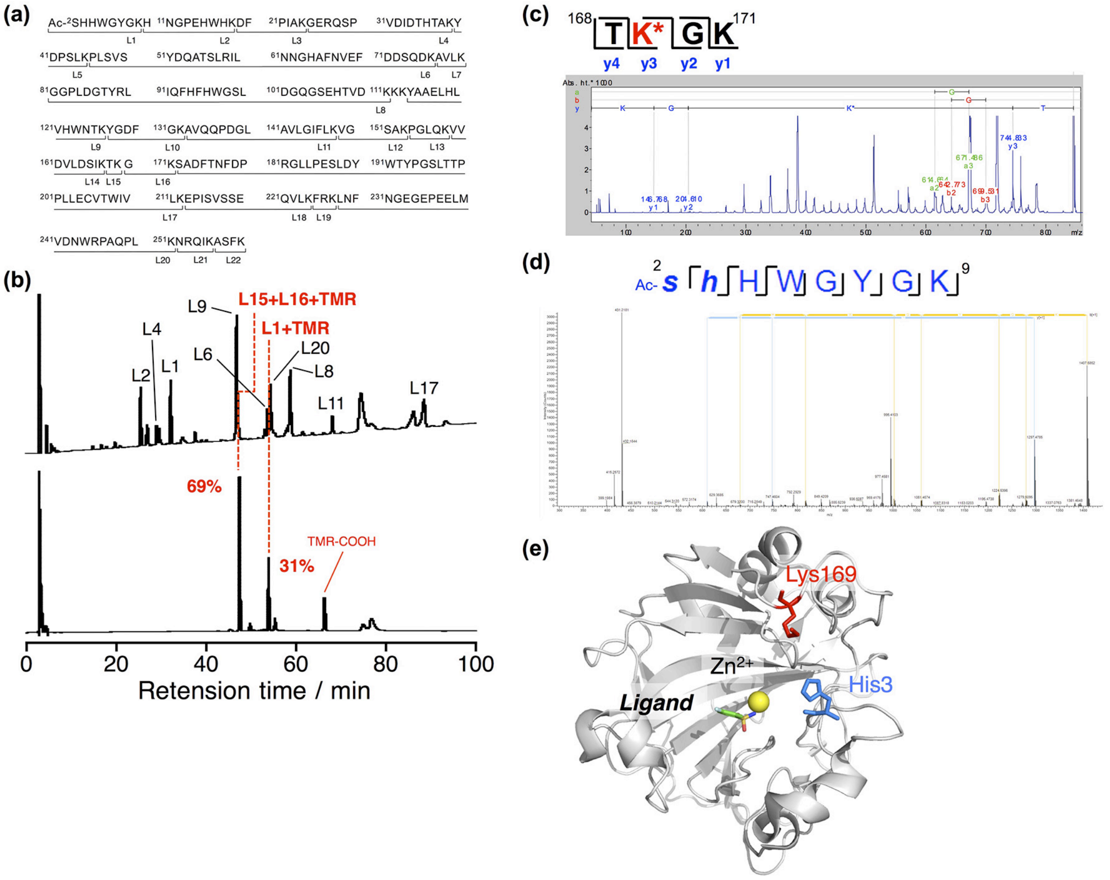

Figure S10. Mass spectral analysis of the labeling site of hCAII by the reaction with LDBB reagent **11**. **(a)** The primary sequence of hCAII and the predictable fragments generated by LysC digestion. (**b**) HPLC analysis of the digested fragments of hCAII. The chromatograms were shown for the fragments derived from the **11**-labeled hCAII detected with UV absorption at 220 nm (top) or 550 nm (bottom). The peaks marked with the character (e.g. L1) correspond to the labeled fragments, which were characterized by MALDI-TOF MS analysis. (**c**) MALDI-TOF MS/MS analysis of the dye-labeled L15+L16+TMR. **(d)** MALDI-Orbitrap MS/MS analysis of L1+TMR. (**e**) 3D structure of hCAII (the labeling site (Lys169 or His3) was shown in red or blue, Zn ion was in yellow, and the ligand in green stick, PDB ID: 1IF4).

Supplementary Methods

Synthesis of reagents 1 – 9 for eDHFR labeling.

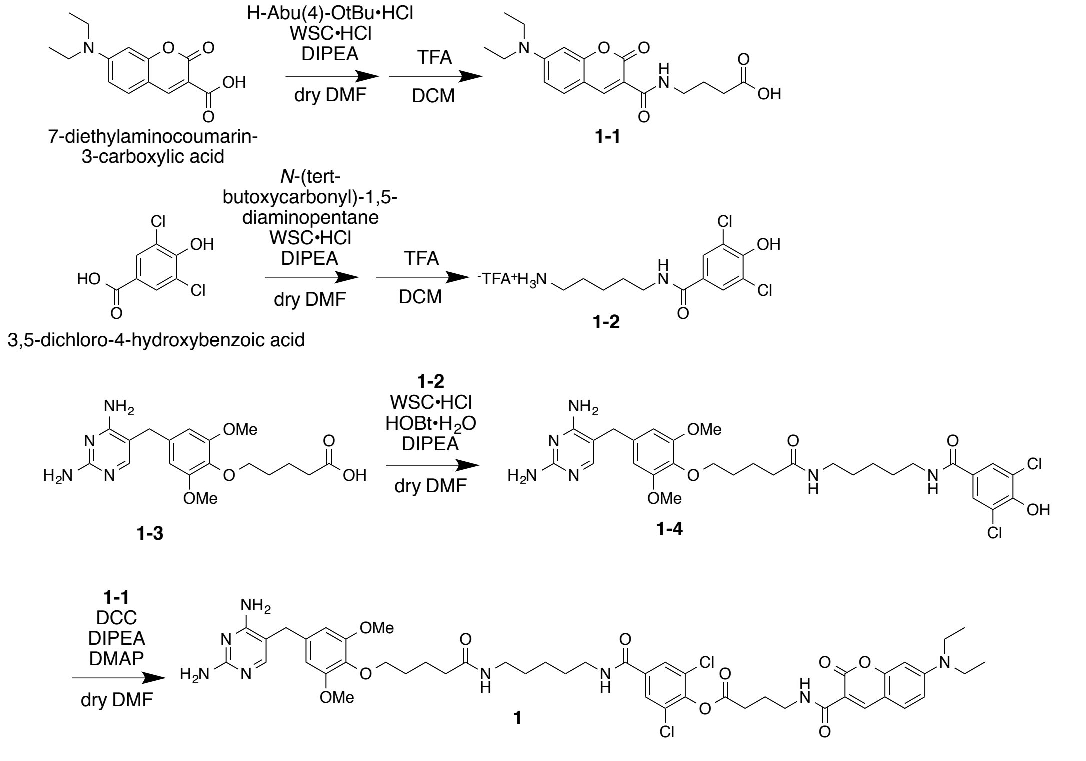

Scheme S1. Synthetic scheme of compounds **1**.

Compound 1-1

To a stirred solution of 7-diethylaminocoumarin-3-carboxylic acid (783 mg, 3.0 mmol) in dry DMF (3.0 mL) was added *N,N*-diisopropylethyl amine (DIPEA) (1.3 mL, 7.5 mmol), water soluble carbodiimide (WSC•HCl) (690 mg, 3.6 mmol) and H-Abu(4)-OtBu•HCl (646 mg, 3.3 mmol). The reaction mixture was allowed to stir at 35ºC for overnight. After evaporation, the residue was purified by column chromatography on silica gel (MeOH : $CHCl_3$ = 1 : 30) and then dissolved in AcOEt (200 mL). The organic solution was washed with sat. $NaHCO_3$ aq (50 mL, twice), 5% citric acid aq. (50 mL, 4 times) and brine (50 mL, twice). After dried over $MgSO_4$, the organic layer was evaporated to yield the target compound (450 mg, 1.1 mmol, 37%) as yellow solid.

To a stirred solution of the above compound (449 mg, 1.1 mmol) in dichloromethane (DCM) (8.0 mL) was added trifluoroacetic acid (TFA) (2.0 mL). The reaction was monitored by TLC (silica, $CHCl_3$: MeOH : AcOH = 10 : 1 : 0.1) to confirm the end of this reaction (over 2 h at r.t.). After co-evaporated with toluene

(2.0 mL) twice, the residue was precipitated with diethylether (2.0 mL), filtered and dried in vacuo to yield compound **1-1** (440 mg, 960 mmol, 86%) as yellow solid. ^{1}H NMR (CD_3OD, 400 MHz, r.t.) δ/ppm = 1.23 (t, J = 7.2 Hz, 6H), 1.90 (m, 2H), 2.38 (t, J = 7.2 Hz, 2H), 3.45 (t, J = 7.2 Hz, 4H), 3.52 (q, J = 7.2 Hz, 4H), 6.57 (m, 1H), 6.82 (m, 1H), 7.55 (d, J = 8.8 Hz, 1H), 8.61 (s, 1H). ^{13}C NMR (CD_3OD, 100 MHz, r.t.) δ/ppm = 12.67, 27.76, 32.13, 39.67, 45.84, 97.13, 109.28, 109.86, 111.41, 111.60, 132.36, 148.11, 154.21, 158.80, 163.92, 176.53. IR (film): ν (cm^{-1}) 3334, 2977, 2937, 1700, 1618, 1580, 1541, 1512, 1457, 1419, 1353, 1238, 1188, 1136, 1079, 792. HR-MS (ESI); calc. for $C_{18}H_{22}N_2O_5Na_1$ $[M+Na]^+$; 369.1426, Found; 369.1392.

Compound 1-2

To a stirred solution of *N*-(tert-butoxycarbonyl)-1,5-diaminopentane (1.1 g, 5.4 mmol) in dry DMF (5.0 mL) was added 3,5-dichloro-4-hydroxybenzoic acid (1.0 g, 4.6 mmol), WSC•HCl (1.1 g, 5.5 mmol) and DIPEA (1.9 mL, 11.0 mmol). The reaction mixture was allowed to stir at r.t. for 6 h and then at 40ºC for 8 h. After evaporated, the residue was roughly purified by column chromatography on silica gel (MeOH : $CHCl_3$ = 1 : 30). Then, the crude was dissolved in AcOEt (100 mL) and washed with H_2O (50 mL, 4 times) and brine (20 mL, once). The organic layer was dried over $MgSO_4$, evaporated and dried in vacuo to yield the target compound (220 mg, 560 mmol, 11%) as colorless oil.

To a stirred solution of the above compound (180 mg, 460 mmol) in DCM (7.0 mL) was added TFA (3.0 mL). The reaction was monitored by TLC (silica, $CHCl_3$: MeOH = 10 : 1) to confirm the end of this reaction (over 3 h at r.t.). After co-evaporated with toluene (2.0 mL) twice, the residue was precipitated with diethylether (2.0 mL), filtered and dried in vacuo to yield compound **1-2** (180 mg, 440 mmol, 96%) as yellow solid. ^{1}H NMR (CD_3OD, 400 MHz, r.t.) δ/ppm = 1.47 (m, 2H), 1.61-1.73 (m, 4H), 2.92 (t, J = 7.2, 2H), 3.36 (t, J = 7.2 Hz, 2H), 7.79 (s, 2H), 7.98 (br, 1H). ^{13}C NMR (CD_3OD, 100 MHz, r.t.) δ/ppm = 25.17, 28.77, 30.07, 30.64, 41.08, 123.25, 128.77, 128.80, 130.43, 153.51, 167.25. IR (film): ν (cm^{-1}) 3321, 3082, 2974, 2938, 2865, 1689, 1637, 1593, 1558, 1485, 1393, 1367, 1169, 1025, 802, 759. HR-MS (ESI); calc. for $C_{12}H_{17}Cl_2N_2O_2$ $[M+H]^+$; 291.0662, Found; 291.0656.

Compound 1-4

To a stirred solution of compound **1-3**[S1] (178 mg, 473 mmol) in dry DMF (3.0 mL) was added 1-hydroxy-1*H*-benzotriazole monohydrate (HOBt•H_2O) (73 mg, 473 mmol), WSC•HCl (99 mg, 516 mmol), DIPEA (0.26 mL, 1.5 mmol) and compound **1-2** (174 mg, 430 mmol). The reaction mixture was allowed to stir at r.t. for 3.5 h. After evaporation, the residue was purified by column chromatography on silica gel (MeOH : $CHCl_3$: AcOH = 1 : 6 : 0.1 to 1 : 4: 0.1). The residue was co-evaporated with toluene and dried in vacuo to yield compound **1-4** (115 mg, 177 mmol, 41%) as white amorphous. ^{1}H NMR (CD_3OD, 400 MHz, r.t.) δ/ppm = 1.51-1.64 (m, 4H), 1.68-1.82 (m, 4H), 2.25 (t, J = 7.2, 2H), 3.19 (t, J = 6.4 Hz, 2H), 3.32 (m, 2H), 3.62 (s, 2H), 3.77 (s, 6H), 3.91 (t, J = 6.0 Hz, 2H), 6.52 (s, 2H), 7.72 (s, 2H). ^{13}C NMR (CD_3OD, 100 MHz, r.t.) δ/ppm = 22.53, 23.71, 25.26, 30.08, 30.53, 34.04, 36.82, 40.15, 40.79, 56.61, 73.92, 107.24, 110.43, 123.59, 124.29, 128.57, 134.13, 137.01, 143.45, 155.03, 157.62, 158.43, 165.86, 168.10, 176.13. IR (film): ν (cm^{-1}) 3382, 2939, 2875, 2843, 1661, 1636, 1592, 1560, 1458, 1437, 1421, 1298, 1227, 1187, 1146, 1123, 1123, 1018, 838. HR-MS (ESI); calc. for $C_{30}H_{38}Cl_2N_6O_6Na_1$ [M+Na]$^+$; 671.2128, Found; 671.2107.

Compound 1

To a stirred solution of compound **1-1** (2.8 mg, 6 μmol) in dry DMF (0.3 mL) was added dicyclohexylcarbodiimide (DCC) (2.4 mg, 14 μmol), DIPEA (2.4 μL, 14 μmol), compound **1-4** (3.2 mg, 5 μmol) and *N,N*-4-dimethylaminopyridine (DMAP) (0.6 mg, 5 μmol). The reaction mixture was allowed to stir at r.t. for 5 h. After evaporation, the residue was purified by column chromatography on silica gel (MeOH : $CHCl_3$: AcOH = 1 : 8 : 0.1 to 1 : 6: 0.1). The residue was co-evaporated with toluene and dried in vacuo to yield compound **1** (2.7 mg, 3 μmol, 47%) as yellow amorphous. ^{1}H NMR (CD_3OD, 400 MHz, r.t.) δ/ppm = 1.22 (t, J = 7.2 Hz, 6H), 1.40 (m, 2H), 1.50-1.64 (m, 4H), 1.68-1.77 (m, 2H), 2.08 (t, J = 7.2, 2H), 2.23 (t, J = 7.2 Hz, 2H), 2.81 (t, J = 7.2 Hz, 2H), 3.48-3.56 (m, 6h), 3.62 (s, 2H), 3.77 (s, 6H), 3.88 (t, J = 6.0 Hz, 2H), 6.52 (s, 2H), 6.81 (m, 1H), 7.55 (d, J = 9.2 Hz, 1H), 7.89 (s, 2H), 8.63 (s, 1H). ^{13}C NMR (DMSO-d_6, 100 MHz, r.t.) δ/ppm = 12.30, 18.54, 19.89, 21.87,

23.80, 28.49, 28.91, 29.19, 32.95, 35.07, 38.25, 38.38, 44.32, 55.83, 72.02, 95.85, 105.85, 105.90, 107.63, 109.41, 110.11, 126.89, 127.71, 127.97, 131.52, 134.34, 134.86, 135.62, 145.11, 147.65, 152.39, 152.85, 157.18, 161.22, 161.77, 162.15, 162.15, 162.22, 162.90, 167.15, 171.85. IR (film): ν (cm^{-1}) 3385, 2939, 2870, 1696, 1684, 1653, 1647, 1636, 1617, 1577, 1559, 1540, 1512, 1457, 1437, 1420, 1353, 1233, 1189, 1127, 1056, 1033, 1017, 771. HR-MS (ESI); calc. for $C_{48}H_{58}Cl_2N_8O_{10}$ $[M+H]^+$; 977.3726, Found; 977.3727.

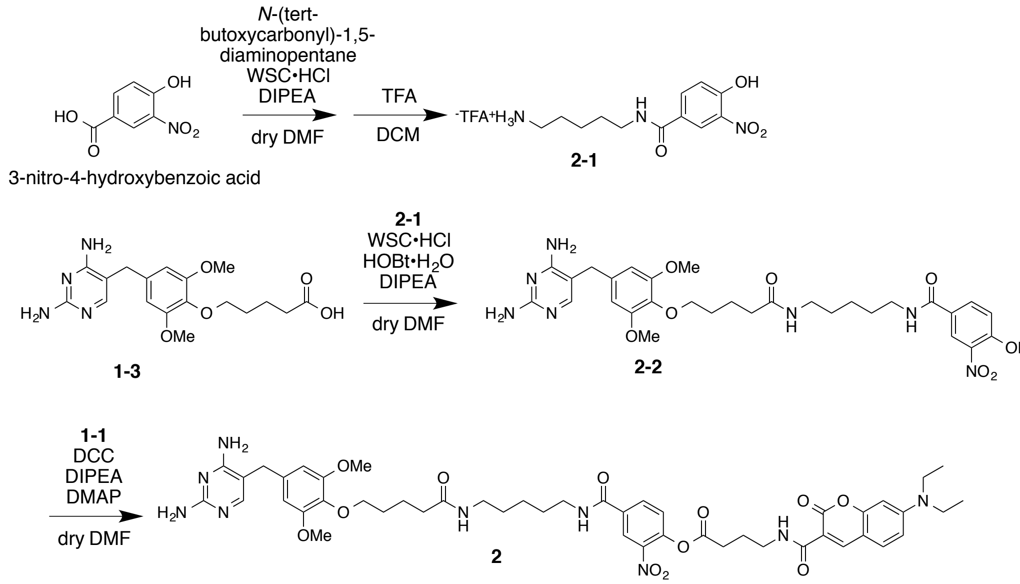

Scheme S2. Synthetic scheme of compounds **2**.

Compound 2-1

To a stirred solution of *N*-(tert-butoxycarbonyl)-1,5-diaminopentane (243 mg, 1.2 mmol) in dry DMF (2.0 mL) was added 3-nitro-4-hydroxybenzoic acid (200 mg, 1.1 mmol), WSC•HCl (288 mg, 1.5 mmol), HOBt•H_2O (167 mg, 1.1 mmol) and DIPEA (0.53 mL, 3.0 mmol). The reaction mixture was allowed to stir at r.t. for 12 h. After evaporated, the residue was dissolved in AcOEt (200 mL) and washed with 5% citric acid aq. (50 mL, twice), sat. $NaHCO_3$ aq. (100 mL, 3 times), H_2O (100 mL, twice) and brine (50 mL, once). The organic layer was dried over $MgSO_4$, evaporated and dried in vacuo to yield the target compound (304 mg, 0.8 mmol, 76%) as yellow solid.

To a stirred solution of the above compound (200 mg, 0.544 mmol) in DCM (8.0 mL) was added TFA (2.0 mL). The reaction was monitored by TLC (silica, $CHCl_3$: MeOH = 10 : 1) to confirm the end of this reaction (over 3 h at r.t.). After co-evaporated with toluene (2.0 mL) twice, and then dried in vacuo to yield compound **2-1** (243 mg, quantitative) as yellow amorphous. ^{1}H NMR (CD_3OD, 400 MHz, r.t.) δ/ppm = 1.46 (m, 2H), 1.64-1.75 (m, 4H), 2.93 (t, J = 7.2, 2H), 3.40 (t, J = 7.2 Hz, 2H), 7.22 (d, J = 8.8 Hz, 1H), 8.05 (m, 1H), 8.57 (m, 1H). ^{13}C NMR (CD_3OD, 100 MHz, r.t.) δ/ppm = 24.76, 28.19, 29.96, 40.54, 40.61, 121.00, 125.79, 127.44, 135.88, 135.98, 157.34, 167.49. IR (film): ν (cm^{-1}) 3286, 3079, 2991, 2951, 2871, 1679, 1627, 1559, 1533, 1486, 1424, 1323, 1258, 1202, 1137, 841, 799, 723. HR-MS (ESI); calc.

for $C_{12}H_{18}N_3O_4$ $[M+H]^+$; 268.1292, Found; 268.1288.

Compound 2-2

To a stirred solution of compound **1-3** (113 mg, 0.30 mmol) in dry DMF (1.0 mL) was added HOBt•H_2O (48 mg, 0.30 mmol), WSC•HCl (63 mg, 0.33 mmol), DIPEA (0.18 mL, 1.0 mmol) and compound **2-1** (122 mg, 0.32 mmol). The reaction mixture was allowed to stir at r.t. for 12 h. After evaporation, the residue was roughly purified by column chromatography on silica gel (MeOH : $CHCl_3$ = 1 : 6). The crude compound was further purified by HPLC (ODS-A, flow 9.999 ml/min, gradient; A (CH_3CN containing 0.1% TFA) : B (H_2O containing 0.1% TFA) = 10 : 90 (0 min) to 30 : 70 (15 min) to 50 : 50 (55 min)) and the obtained solution was lyophilized to yield compound **2-2** (137 mg, 0.22 mmol, 73%) as yellow amorphous. ^{1}H NMR (CD_3OD, 400 MHz, r.t.) δ/ppm = 1.41 (m, 2H), 1.52-1.80 (m, 8H), 2.24 (t, J = 7.2, 2H), 3.19 (t, J = 6.8 Hz, 2H), 3.37 (t, J = 7.2 Hz, 2H), 3.65 (s, 2H), 3.79 (s, 6H), 3.89 (t, J = 6.0 Hz, 2H), 6.54 (s, 2H), 7.18-7.21 (m, 2H), 8.05 (m, 1H), 8.56 (m, 1H). ^{13}C NMR (CD_3OD, 100 MHz, r.t.) δ/ppm = 23.71, 25.31, 30.05, 30.11, 30.58, 33.92, 36.81, 40.16, 40.97, 56.64, 73.92, 105.03, 107.27, 110.03, 120.97, 125.77, 133.67, 135.37, 135.80, 136.03, 137.21, 140.39, 155.09, 156.18, 157.30, 166.29, 167.39, 176.10. IR (film): ν (cm^{-1}) 3286, 2972, 2945, 2931, 2866, 2836, 1665, 1646, 1637, 1595, 1528, 1507, 1475, 1349, 1323, 1249, 1201, 1161, 1131, 1028, 840, 800, 771, 759. 734, 722, 703. HR-MS (ESI); calc. for $C_{30}H_{40}N_7O_8$ $[M+H]^+$; 626.2933, Found; 626.2975.

Compound 2

To a stirred solution of compound **2-1** (22 mg, 48 μmol) in dry DMF (0.4 mL) was added DCC (15 mg, 60 μmol), DIPEA (14 μL, 104 μmol), compound **2-2** (29 mg, 46 μmol) and DMAP (5 mg, 40 μmol). The reaction mixture was allowed to stir at r.t. for 12 h. After evaporation, the residue was purified by column chromatography on silica gel (MeOH : $CHCl_3$ = 1 : 7). The residue was re-precipitated with diethylether (2.0 mL), filtered and dried in vacuo to yield compound **2** (3 mg, 3 μmol, 7%) as yellow amorphous. ^{1}H NMR (DMSO-d_6, 400 MHz, r.t.) δ/ppm = 1.09 (t, J = 7.2 Hz, 6H), 1.24 (m, 2H), 1.37 (m, 2H), 1.44-1.57 (m, 6H), 1.86 (m, 2H), 2.03 (t, J = 6.8,

2H), 2.67 (t, J = 7.2 Hz, 2H), 2.98 (m, 2H), 3.65 (s, 6H), 3.71 (t, J = 6.0 Hz, 2H), 5.77 (s, 2H), 6.18 (s, 2H), 6.48 (s, 2H), 6.55 (m, 1H), 6.75 (m, 1H), 7.45 (s, 1H), 7.54 (d, J = 8.4 Hz, 1H), 7.62 (d, J = 9.2 Hz, 1H), 7.71 (br, 1H), 8.19 (m, 1H), 8.52 (m, 1H), 8.60 (m, 1H) 8.70 (br-t, J = 5.6 Hz, 1H), 8.75 (br-t, J = 5.6 Hz, 1H). ^{13}C NMR (DMSO-d_6, 100 MHz, r.t.) δ/ppm = 12.30, 18.54, 20.53, 21.86, 23.84, 28.55, 29.04, 29.19, 32.94, 35.06, 38.27, 44.32, 55.83, 72.02, 95.84, 105.86, 107.63, 109.40, 110.10, 110.13, 124.41, 125.49, 131.53, 133.27, 133.91, 134.87, 135.59, 141.22, 144.91, 147.66, 152.40, 152.85, 157.19, 161.78, 162.16, 162.25, 163.31, 168.30, 171.83. IR (film): ν (cm^{-1}) 3444, 1640, 1577, 1558, 1511, 1419, 1350, 1233, 1206, 1189, 1135, 1189, 1135, 1079, 1056, 1021. HR-MS (ESI); calc. for $C_{48}H_{59}N_9O_{12}$ [M+H]$^+$; 954.4356, Found; 954.4384.

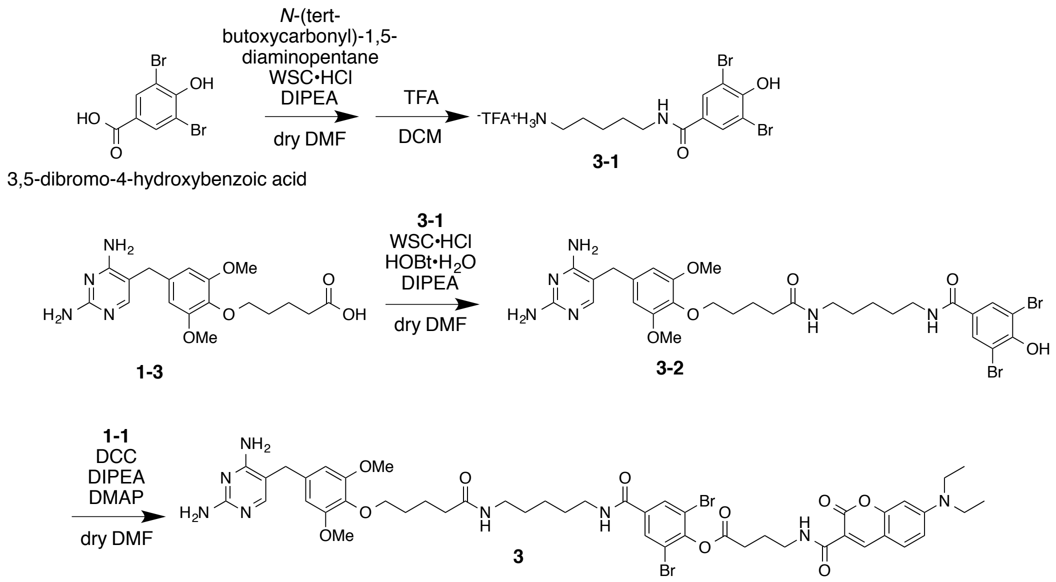

Scheme S3. Synthetic scheme of compounds **3**.

Compound 3-1

To a stirred solution of *N*-(tert-butoxycarbonyl)-1,5-diaminopentane (890 mg, 4 mmol) in dry DMF (6.0 mL) was added 3,5-dibromo-4-hydroxybenzoic acid (1.2 g, 4 mmol), WSC•HCl (1.1 g, 5.6 mmol), HOBt•H_2O (612 mg, 4.0 mmol) and DIPEA (1.8 mL, 10 mmol). The reaction mixture was allowed to stir at r.t. for 12 h. After evaporated, the residue was dissolved in AcOEt (200 mL) and washed with 5% citric acid aq. (50 mL, twice), H_2O (50 mL, twice) and brine (50 mL, once). The organic layer was dried over $MgSO_4$, evaporated and dried in vacuo to yield the target compound (1.7 g, 3.5 mmol, 88%) as white amorphous.

To a stirred solution of the above compound (480 mg, 1.0 mmol) in DCM (7.0 mL) was added TFA (3.0 mL). The reaction was monitored by TLC (silica, $CHCl_3$: MeOH = 10 : 1) to confirm the end of this reaction (over 3 h at r.t.). After co-evaporated with toluene (2.0 mL) twice. The residue was re-precipitated with diethylether (2.0 mL), evaporated and then dried in vacuo to yield compound **2-1** (483 mg, 0.98 mmol, 98%) as yellow solid. ^{1}H NMR (CD_3OD, 400 MHz, r.t.) δ/ppm = 1.46 (m, 2H), 1.63-1.75 (m, 4H), 2.93 (t, J = 7.2, 2H), 3.41 (t, J = 7.2 Hz, 2H), 8.15 (s, 2H). ^{13}C NMR (CD_3OD, 100 MHz, r.t.) δ/ppm = 24.73, 28.18, 29.94, 40.54, 40.61, 111.85, 129.15, 132.68, 155.31, 167.11. IR (film): ν (cm^{-1}) 3080, 2951, 2847, 1677, 1635, 1548, 1474, 1438, 1395, 1304, 1204, 1139, 723. HR-MS (ESI); calc. for $C_{12}H_{17}Br_2N_2O_2$ $[M+H]^+$; 378.9651, Found; 378.9647.

Compound 3-2

To a stirred solution of compound **1-3** (207 mg, 0.55 mmol) in dry DMF (3.0 mL) was added HOBt•H_2O (84 mg, 0.55 mmol), WSC•HCl (115 mg, 0.6 mmol), DIPEA (0.32 mL, 1.8 mmol) and compound **3-1** (247 mg, 0.50 mmol). The reaction mixture was allowed to stir at r.t. for 12 h. After evaporation, the residue was roughly purified by column chromatography on silica gel (MeOH : $CHCl_3$: AcOH = 1 : 6 : 0.1). The crude compound was further purified by HPLC (OSD-A, flow 9.999 ml/min, gradient; A (CH_3CN containing 0.1% TFA) : B (H_2O containing 0.1% TFA) = 10 : 90 (0 min) to 30 : 70 (15 min) to 50 : 50 (55 min)) and the obtained solution was lyophilized to yield compound **3-2** (47 mg, 0.063 mmol, 13%) as brown oil. ^{1}H NMR (CD_3OD, 400 MHz, r.t.) δ/ppm = 1.38 (m, 2H), 1.52-1.65 (m, 4H), 1.68-1.82 (m, 4H), 2.24 (t, J = 7.2, 2H), 3.18 (t, J = 6.8 Hz, 2H), 3.33 (m, 2H), 3.65 (s, 2H), 3.79 (s, 6H), 3.90 (t, J = 6.0 Hz, 2H), 6.52 (s, 2H), 7.21 (s, 1H), 7.98 (s, 2H). ^{13}C NMR (CD_3OD, 100 MHz, r.t.) δ/ppm = 23.68, 25.24, 29.95, 30.05, 30.53, 33.91, 36.77, 40.15, 40.92, 56.63, 73.90, 107.22, 110.90, 111.82, 129.22, 132.66, 133.65, 137.08, 140.38, 155.01, 155.13, 156.14, 166.18, 166.93, 176.07. IR (film): ν (cm^{-1}) 3322, 3314, 3209, 2942, 2868, 1661, 1633, 1592, 1575, 1544, 1505, 1460, 1296, 1244, 1202, 1183, 1127, 1069, 1029, 836, 801, 769, 722. HR-MS (ESI); calc. for $C_{30}H_{39}Br_2N_6O_6$ $[M+H]^+$; 737.1292, Found; 737.1326.

Compound 3

To a stirred solution of compound **3-1** (11 mg, 24 μmol) in dry DMF (0.4 mL) was added DCC (5 mg, 24 μmol), DIPEA (7.2 μL, 52 μmol), compound **3-2** (15 mg, 20 μmol) and DMAP (2 mg, 20 μmol). The reaction mixture was allowed to stir at r.t. for 3 h, and then stir at 50ºC for 3 h. After evaporation, the residue was purified by column chromatography on silica gel (MeOH : $CHCl_3$ = 1 : 7). The residue was re-precipitated with diethylether (2.0 mL), filtered and dried in vacuo to yield compound **3** (12 mg, 12 μmol, 58%) as yellow amorphous. ^{1}H NMR (CD_3OD, 400 MHz, r.t.) δ/ppm = 1.21 (t, J = 7.2 Hz, 6H), 1.39 (m, 2H), 1.51-1.78 (m, 8H), 2.08 (t, J = 7.2, 2H), 2.22 (t, J = 7.2 Hz, 2H), 2.80 (t, J = 7.2 Hz, 2H), 3.17 (t, J = 6.8 Hz, 2H), 3.34 (m, 2H), 3.48-3.57 (m, 6H), 3.61 (s, 2H), 3.75 (s, 6H), 3.87 (t, J = 6.0 Hz, 2H), 6.49 (s,

2H), 6.55 (s, 1H), 6.81 (m, 1H), 7.45 (s, 1H), 7.53 (d, J = 9.2 Hz, 1H), 8.07 (s, 2H), 8.60 (s, 2H). ^{13}C NMR (CD_3OD, 100 MHz, r.t.) δ/ppm = 12.69, 23.72, 25.24, 25.74, 29.86, 32.02, 34.04, 39.73, 40.10, 41.10, 45.99, 97.28, 107.21, 109.48, 110.11, 110.47, 111.69, 118.94, 132.54, 132.67, 136.10, 137.14, 148.00, 149.36, 149.84, 154.63, 155.05, 159.19, 164.09, 165.65, 165.92, 166.26, 170.61, 176.12. IR (film): ν (cm^{-1}) 3335, 3078, 2933, 2869, 1773, 1684, 1664, 1635, 1617, 1581, 1562, 1540, 1512, 1457, 1419, 1380, 1351, 1315, 1255, 1233, 1201, 1188, 1132, 1082, 1023, 1012, 835, 801, 751, 719, 710. HR-MS (ESI); calcd. for $C_{48}H_{58}Br_2N_8O_{10}$ $[M+H]^+$; 1065.2715, Found; 1065.2712.

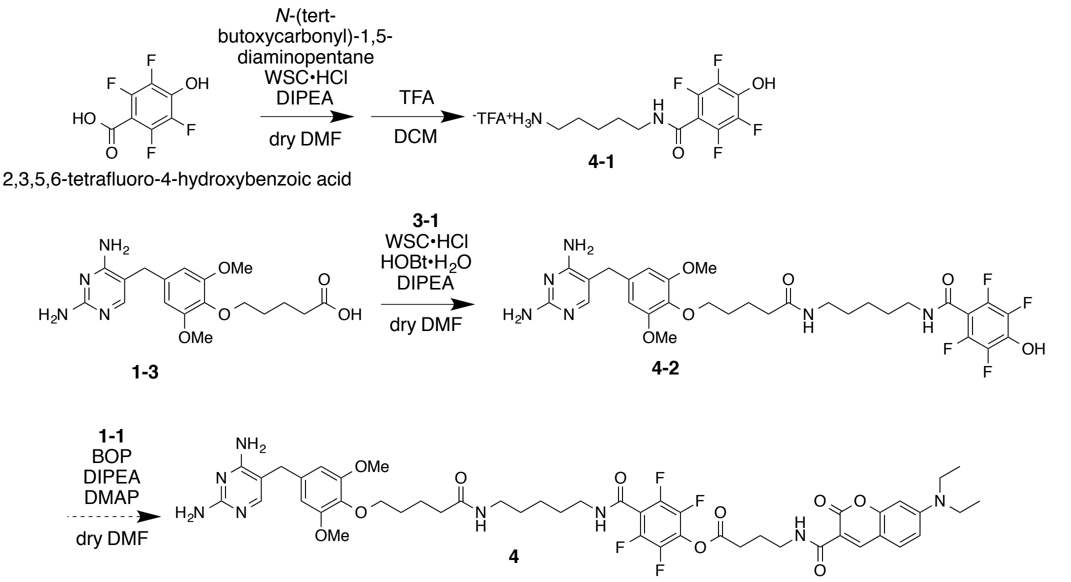

Scheme S4. Synthetic scheme of compounds **4** (final compound could not be isolated).

Compound 4-1

To a stirred solution of *N*-(tert-butoxycarbonyl)-1,5-diaminopentane (1.0 g, 5.0 mmol) in dry DMF (14 mL) was added 2,3,5,6-tetrafluoro-4-hydroxybenzoic acid (966 mg, 4.6 mmol), WSC•HCl (1.1 g, 5.5 mmol) and DIPEA (1.9 mL, 11 mmol). The reaction mixture was allowed to stir at 45ºC for 12 h. After evaporated, the residue was dissolved in AcOEt (200 mL) and washed with 5% citric acid aq. (50 mL, 3 times), H_2O (20 mL, once) and brine (20 mL, once). The organic layer was dried over $MgSO_4$, evaporated and dried in vacuo to yield the target compound (540 mg, 1.4 mmol, 30%) as brown oil.

To a stirred solution of the above compound (540 mg, 1.4 mmol) in DCM (5.0 mL) was added TFA (5.0 mL). The reaction was monitored by TLC (silica, $CHCl_3$: MeOH = 10 : 1) to confirm the end of this reaction (over 4 h at r.t.). After co-evaporated with toluene (2.0 mL) twice and then dried in vacuo to yield compound **4-1** (479 mg, 1.17 mmol, 87%) as brown oil. ^{1}H NMR (CD_3OD, 400 MHz, r.t.) δ/ppm = 1.48 (m, 2H), 1.62-1.72 (m, 4H), 2.93 (t, J = 7.6, 2H), 3.38 (t, J = 5.6 Hz, 2H).

Compound 4-2

To a stirred solution of compound **1-3** (207 mg, 0.55 mmol) in dry DMF (3.0 mL) was added WSC•HCl (114 mg, 0.60 mmol), DIPEA (0.31 mL, 1.8 mmol) and compound **4-1** (204 mg, 0.50 mmol). The reaction mixture was allowed to stir at r.t.

for 12 h. After evaporation, the residue was purified by HPLC (OSD-A, flow 9.999 ml/min, gradient; A (CH_3CN containing 0.1% TFA) : B (H_2O containing 0.1% TFA) = 10 : 90 (0 min) to 30 : 70 (15 min) to 50 : 50 (55 min)) and the obtained solution was lyophilized to yield compound **4-2** (198 mg, 0.303 mmol, 61%) as colorless oil. ^{1}H NMR (CD_3OD, 400 MHz, r.t.) δ/ppm = 1.29 (m, 2H), 1.35-1.49 (m, 4H), 1.57-1.61 (m, 4H), 2.07 (m, 2H), 3.00 (m, 2H), 3.18 (m, 2H), 3.94 (s, 2H), 6.58 (s, 2H), 7.38 (s, 1H), 7.51 (s, 2H), 7.74 (s, 2H), 8.28 (br, 1H), 8.66 (br-t, J = 6.0 Hz, 1H), 11.58 (br-s, 1H).

Compound 4 *(this compound could not be isolated)*

To a stirred solution of compound **4-1** (55 mg, 120 μmol) in dry DMF (1.0 mL) was added BOP (62 mg, 140 μmol), DIPEA (60 μL, 340 μmol), compound **4-2** (65 mg, 100 μmol) and DMAP (1 mg, 10 mmol). The reaction mixture was allowed to stir at r.t. for 2 h and then stir at 50ºC for 1 h The reaction monitored by TLC (silica, CHCl3 : MeOH : AcOH = 10 : 1 : 0.1) and MALDI-TOF MS to confirm about 20% of this reaction was proceeded. However, both with column chromatography on silica gel and HPLC, this compound unfortunately decomposed under these purification steps due to the low stability in methanol or water.

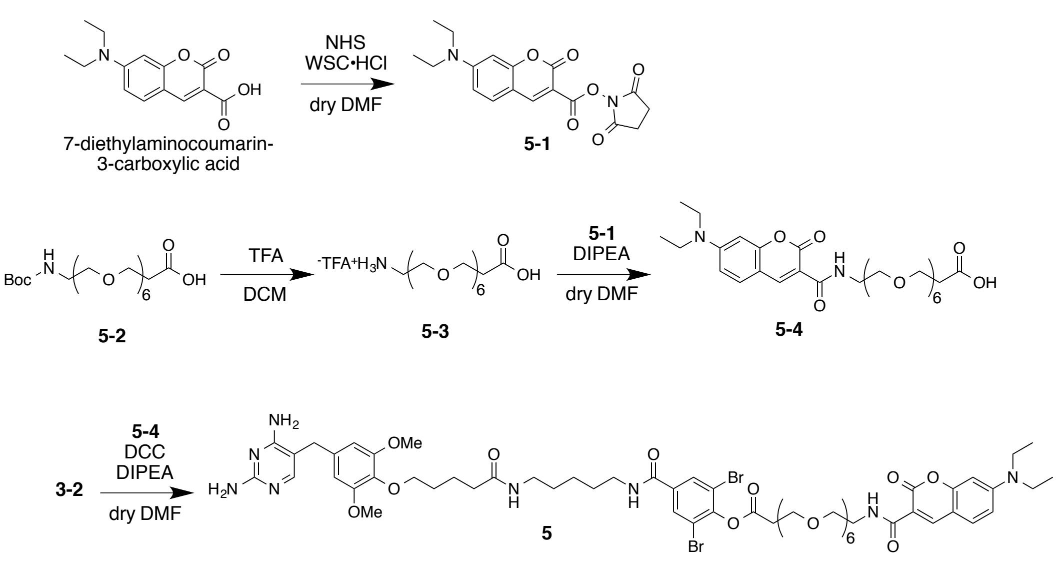

Scheme S4. Synthetic scheme of compounds **5**.

Compound **5-1** was prepared as previously reported.[S2]

Compound 5-4

To a stirred solution of compound **5-2** (commercially available) (90 mg, 198 μmol) in DCM (6.0 mL) was added TFA (2.0 mL). The reaction was monitored by TLC (silica, $CHCl_3$: MeOH = 10 : 1) to confirm the end of this reaction (over 1.5 h at r.t.). After co-evaporated with toluene (1.0 mL) twice and then dried in vacuo to yield compound **5-3** without further purification.

To a stirred solution of the residue of compound **5-3** in dry DMF (2.0 mL) was added DIPEA (52 μL, 297 μmol) and compound **5-1** (85 mg, 238 μmol). The reaction mixture was allowed to stir at r.t. for 4 h. After evaporation, the residue was purified by column chromatography on silica gel ($CHCl_3$: MeOH = 50 : 1 to 30 : 1) to yield compound **5-4** (96 mg, 161 μmol, 81%) as yellow oil. ^{1}H NMR (CD_3OD, 400 MHz, r.t.) δ/ppm = 1.23 (t, J = 6.8 Hz, 6H), 2.53 (t, J = 6.0 Hz, 2H), 3.43-3.75 (m, 26H), 30H), 6.59 (m, 1H), 6.82 (m, 1H), 7.56 (d, J = 9.2 Hz, 1H), 8.64 (s, 1H). This intermediate compound was used for next step without further characterization except for ^{1}H NMR.

Compound 5

To a stirred solution of compound **5-4** (15 mg, 20 μmol) in dry DMF (0.5 mL)

was added DCC (10 mg, 48 μmol), DIPEA (40 μL, 230 μmol), compound **3-2** (23 mg, 30 μmol). The reaction mixture was allowed to stir at r.t. for 43 h. After evaporation, the residue was purified by HPLC (ODS-A, flow 9.999 ml/min, gradient; A (CH_3CN containing 0.1% TFA) : B (H_2O containing 0.1% TFA) = 8 : 92 (0 min) to 82 : 18 (40 min), the target was eluted at 15 min) and the obtained solution was lyophilized to yield compound **5** (14 mg, 10 μmol, 50%) as yellow oil. ^{1}H NMR (CD_3OD, 400 MHz, r.t.) δ/ppm = 1.20 (t, J = 6.8 Hz, 6H), 1.38-1.68 (m, 8H), 2.23 (t, J = 6.0 Hz, 2H), 2.92 (m, 2H), 3.18 (m, 2H), 3.50 (q, J = 6.8 Hz, 4H), 3.59-3.63 (m, 28H), 3.76 (s, 6H), 3.87 (m, 2H), 6.53 (m, 3H), 6.78 (m, 1H), 7.21 (s, 1H), 7.50 (d, J = 8.8 Hz, 1H), 8.05 (s, 2H), 8.58 (s, 1H). ^{13}C NMR (CD_3OD, 100 MHz, r.t.) δ/ppm = 12.74, 23.73, 25.26, 26.13, 29.87, 30.08, 33.94, 35.75, 36.82, 40.11, 40.56, 41.11, 46.00, 56.67, 67.18, 70.59, 71.53, 71.57, 71.60, 73.94, 97.27, 107.30, 109.44, 110.11, 111.01, 111.64, 118.93, 132.56, 132.66, 133.63, 136.06, 137.24, 140.45, 149.29, 149.78, 154.56, 155.09, 156.11, 159.13, 163.90, 165.34. 166.14, 166.26, 169.17, 173.47, 176.10. IR (film): ν (cm^{-1}) 3328, 3210, 2933, 2871, 1772, 1762, 1700, 1653, 1646, 1635, 1617, 1575, 1559, 1550, 1539, 1534, 1521, 1507, 1472, 1457, 1419, 1352, 1203, 1188, 1133, 776. HR-MS (ESI); calcd. for $C_{59}H_{80}Br_2N_8O_{16}$ [M+H]$^+$; 1315.4132, found; 1315.4148.

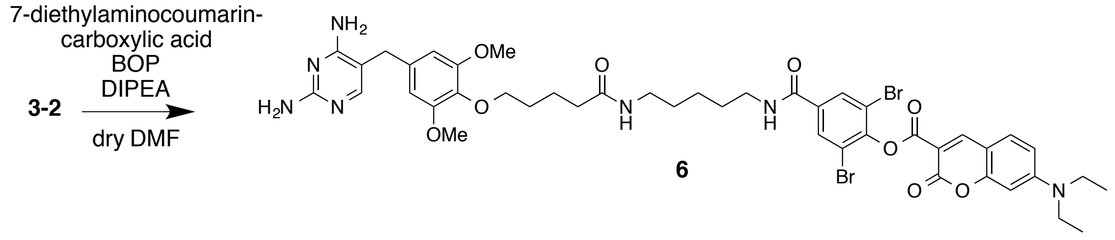

Scheme S5. Synthetic scheme of compound **6**.

Compound 6

To a stirred solution of 7-diethylaminocoumarin-3-carboxylic acid (6 mg, 23 μmol) in dry DMF (0.2 mL) was added BOP (13 mg, 30 μmol), DIPEA (12 μL, 69 μmol) and compound **3-2** (17 mg, 23 μmol). The reaction mixture was allowed to stir at r.t. for 10 h. After evaporation, the residue was purified by HPLC (ODS-A, flow 9.999 ml/min, gradient; A (CH_3CN containing 0.1% TFA) : B (H_2O containing 0.1% TFA) = (1st try); 30 : 70 (0 min) to 85 : 15 (50 min), (2nd try); 40 : 60 (0 min) to 75 : 25 (50 min), the target was eluted at 32 min) and the obtained solution was lyophilized to yield compound **6** (14 mg, 14 μmol, 61%) as yellow oil. ^{1}H NMR (CD_3OD, 400 MHz, r.t.) δ/ppm = 1.26 (t, J = 7.2 Hz, 6H), 1.41-1.79 (m, 13H), 2.25 (t, J = 7.2 Hz, 2H), 3.58 (q, J = 7.2 Hz, 4H), 3.64 (s, 2H), 3.78 (s, 6H), 3.91 (t, J = 6.0 Hz, 2H), 6.53 (s, 2H), 6.60 (m, 1H), 6.88 (d, J = 8.8 Hz, 1H), 7.27 (s, 1H), 7.62 (m, 1H), 8.13 (s, 2H), 8.89 (s, 1H). ^{13}C NMR (CD_3OD, 100 MHz, r.t.) δ/ppm = 12.68, 23.72, 25.27, 29.87, 30.07, 30.56, 36.84, 40.12, 41.14, 46.24, 56.62, 73.95, 97.40, 107.04, 107.26, 109.11, 109.24, 112.02, 119.34, 132.28, 132.58, 133.03, 133.65, 136.12, 136.92, 149.96, 152.69, 155.02, 160.00, 160.27, 160.40, 161.19, 163.58, 166.29, 169.11, 169.60, 176.13. IR (film): ν (cm^{-1}) 3385, 2930, 1762, 1684, 1654, 1647, 1636, 1617, 1582, 1560, 15552, 1544, 1508, 1473, 1457, 1421, 1380, 1352, 1219, 1192, 1157, 1131, 1071, 1033, 1025, 1012, 944, 829, 772, 719. HR-MS (ESI); calcd. for $C_{44}H_{51}Br_2N_7O_9$ [M+H]$^+$; 980.2188, found; 980.2158.

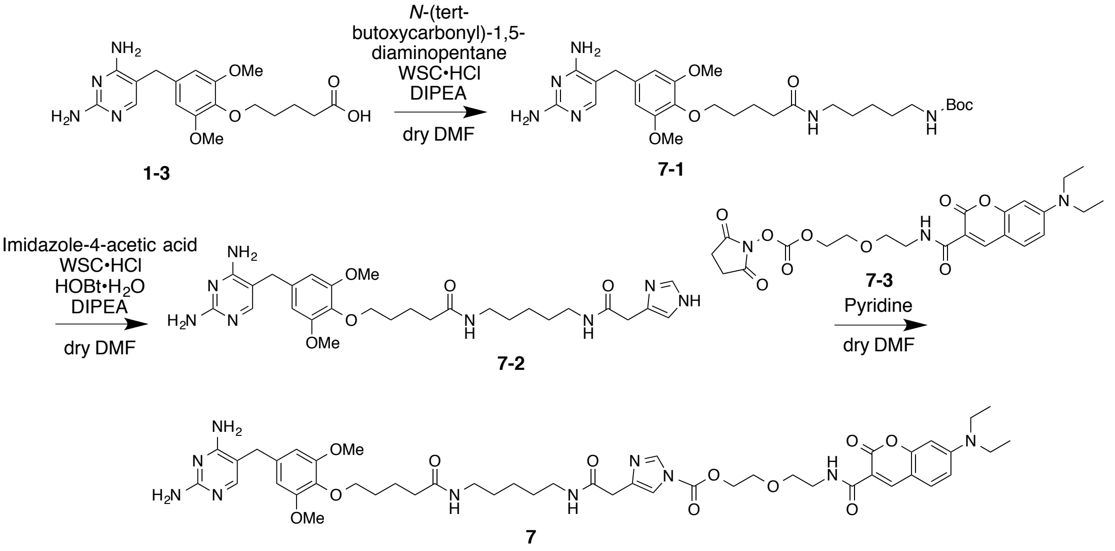

Scheme S6. Synthetic Scheme of compounds **7**.

Compound 7-1

To a stirred solution of compound **1-3** (188 mg, 0.5 mmol) in dry DMF (2.0 mL) was added WSC•HCl (115 mg, 0.6 mmol), HOBt•H_2O (77 mg, 0.5 mmol) and DIPEA (261 μL, 1.5 mmol). The reaction mixture was allowed to stir at 45°C for 8 h. After evaporation, the residue was purified by column chromatography on silica gel ($CHCl_3$: MeOH = 10 : 1) to yield compound **7-1** (220 mg, 0.39 mmol, 78%) as brown oil. ^{1}H NMR (CD_3OD, 400 MHz, r.t.) δ/ppm = 1.33 (m, 2H), 1.41-1.53 (m, 15H), 1.66-1.83 (m, 4H), 2.24 (t, J = 7.2 Hz, 2H), 3.01 (t, J = 7.2 Hz, 2H), 3.15 (t, J = 6.8 Hz, 2H), 3.63 (s, 2H), 3.77 (s, 6H), 3.9(m, 2H), 6.51 (s, 2H), 7.44 (s, 1H).

Compound 7-2

To a stirred solution of compound **7-1** (200 mg, 0.36 mmol) in DCM (4.0 mL) was added TFA (2.0 mL). The reaction was monitored by TLC (silica, $CHCl_3$: MeOH : NH_3 aq. = 5 : 1 : drops) to confirm the end of this reaction (over 6 h at r.t.). After co-evaporated with toluene (2.0 mL) twice and then dried in vacuo to yield compound **7-1** without further purification.

To a stirred solution of the residue of above compound (180 mg, 0.32 mmol) in dry DMF (2.0 mL) was added WSC•HCl (72 mg, 0.38 mmol), HOBt•H_2O (48 mg, 0.32 mmol) and DIPEA (219 μL, 1.3 mmol). The reaction was monitored by TLC (silica, $CHCl_3$: MeOH = 6 : 1) to confirm the end of this reaction (over 16 h at r.t.).

After evaporation, the residue was purified by HPLC (ODS-A, flow 9.999 ml/min, gradient; A (CH_3CN containing 0.1% TFA) : B (H_2O containing 0.1% TFA) = 0 : 100 (0 to 10 min) to 40 : 60 (10 to 40 min) to yield compound **7-2** (68 mg, 0.12 mmol, 38%, retention time; 33 min) as brown oil. ^{1}H NMR (CD_3OD, 400 MHz, r.t.) δ/ppm = 1.23 (m, 2H), 1.48-1.58 (m, 4H), 1.69-1.82 (m, 4H), 2.25 (t, J = 7.2 Hz, 2H), 3.15-3.22 (m, 4H), 3.66 (s, 2H), 3.71 (s, 2H), 3.79 (s, 6H), 3.91 (t, J = 6.0 Hz, 2H), 6.56 (s, 2H), 7.23 (s, 1H), 7.38 (s, 1H), 8.81 (s, 1H).

***Compound** 7*

To a stirred solution of **7-2** (14 mg, 0.25 mmol) in dry DMF (2.0 mL) was added pyridine (20 μL, 20 μmol), compound **7-3**[S3] (0.5 mg, 4 μmol). The reaction was monitored by TLC (silica, $CHCl_3$: MeOH = 10 : 1) to confirm the end of this reaction (50% reaction could be confirmed, over 5 h at r.t.). The reaction mixture was allowed to stir at r.t. for 5.0 h. After evaporation, the residue was purified by column chromatography on silica gel ($CHCl_3$: MeOH = 10 : 1 to 6 : 1) to yield compound **8** (1.5 mg, 1.6 μmol, 1%) as yellow amorphous. ^{1}H NMR (CD_3OD, 400 MHz, r.t.) δ/ppm = 1.23 (t, J = 7.2, 6H), 1.48-1.50 (m, 4H), 1.68-1.78 (m, 4H), 2.23 (t, J = 6.0 Hz, 2H), 3.11-3.23 (m, 4H), 3.44-3.63 (m, 10H), 3.69 (m, 2H), 3.76 (s, 6H), 3.85-3.90 (m, 4H), 6.50 (s, 2H), 6.56 (s, 1H), 6.80 (d, J = 11.2 Hz, 1H), 7.41 (s, 1H), 7.48^7.53 (m, 2H), 8.22 (s, 1H), 8.58 (s, 1H). HR-MS (ESI); calcd. for $C_{47}H_{62}N_{10}O_{11}$ $[M+H]^+$; 943.4672, found; 943.4644.

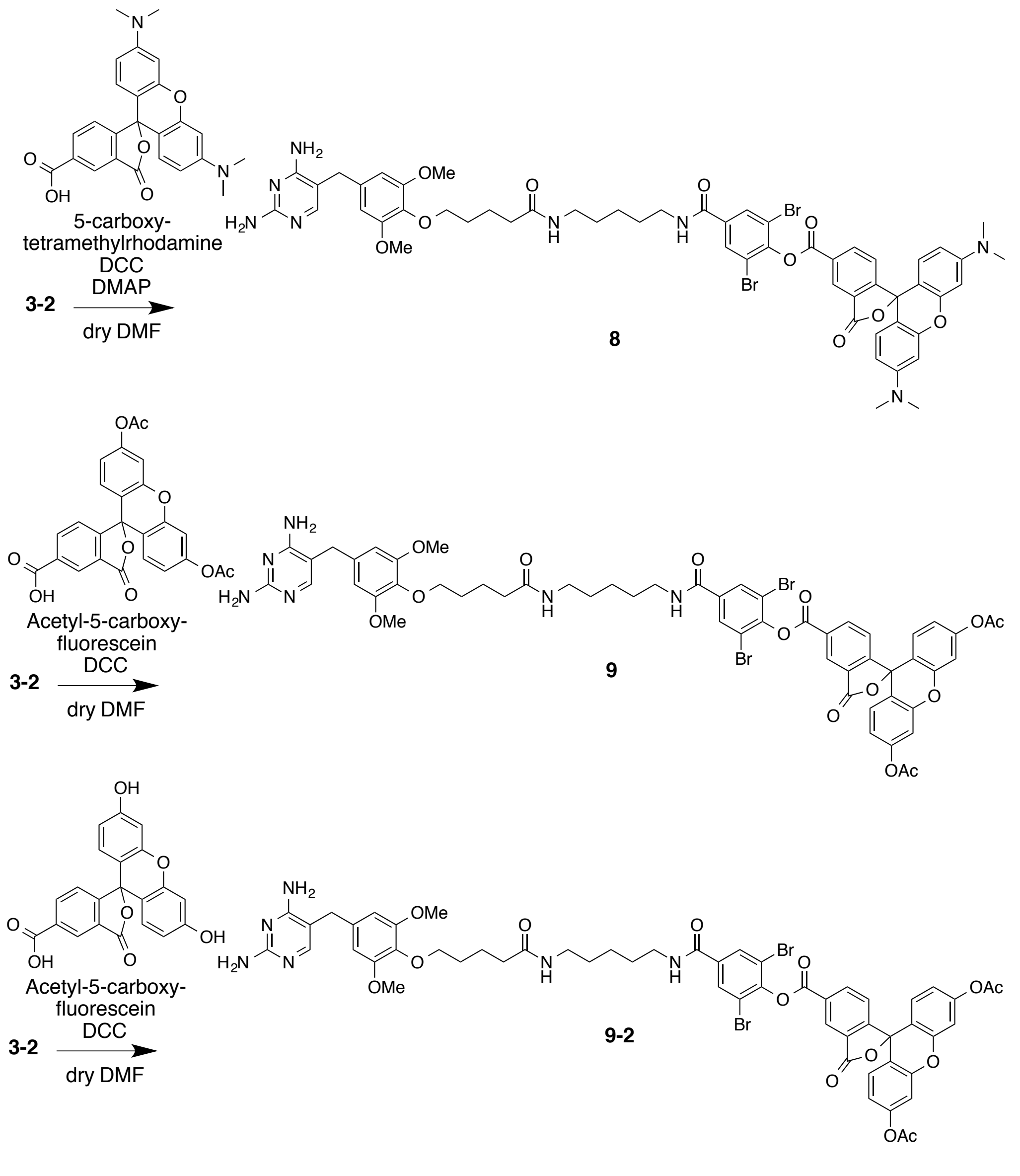

Scheme S7. Synthetic scheme of compounds **8**, **9** and **9-2**.

Compound 8

To a stirred solution of 5-carboxy-tetramethylrhodamine (5.9 mg, 14 μmol) in dry DMF (2.0 mL) was added DCC (4.2 mg, 20 μmol), DMAP (0.5 mg, 4 μmol) and compound **3-2** (10 mg, 14 μmol) at 0ºC. The reaction mixture was allowed to stir at r.t. for 16 h. After evaporation, the residue was purified by HPLC (ODS-A, flow 9.999 ml/min, gradient; A (CH_3CN containing 0.1% TFA) : B (H_2O containing 0.1% TFA) = 30 : 70 (0 min) to 80 : 20 (50 min)) and the obtained solution was lyophilized to yield compound **8** (2.8 mg, 2.4 μmol, 18%) as purple solid. ^{1}H NMR (CD_3OD, 400 MHz, r.t.) δ/ppm = 1.44 (m, 2H), 1.53-1.84 (m, 8H), 2.27 (t, J = 7.2 Hz, 2H), 3.22 (t,

J = 7.2 Hz, 2H), 3.30 (s, 12H), 3.40 (m, 2H), 3.66 (s, 2H), 3.81 (s, 6H), 3.93 (t, J = 6.4 Hz, 2H), 6.57 (s, 2H), 7.02 (d, J = 2.0 Hz, 2H), 7.09 (dd, J = 9.6, 2.4 Hz, 2H), 7.18 (s, 1H), 7.21 (d, J = 9.6 Hz, 2H), 7.72 (d, J = 8.0 Hz, 1H), 8.23 (s, 2H), 8.66 (dd, J = 8.0, 2.0 Hz, 1H), 9.11 (br, 1H). ^{13}C NMR (CD_3OD, 100 MHz, r.t.) δ/ppm = 23.73, 29.89, 30.13, 30.61, 33.94, 36.84, 40.16, 40.93, 40.97, 41.19, 56.66, 73.97, 87.62, 97.53, 107.30, 111.00, 114.55, 115.71, 118.88, 131.23, 131.88, 132.81, 133.68, 134.08, 135.06, 137.22, 140.39, 141.21, 143.81, 144.94, 149.65, 155.11, 156.20, 159.00, 159.70, 162.77, 166.27, 167.18, 168.36, 176.24. IR (film): ν (cm^{-1}) 3344, 3220, 2946, 2872, 1755, 1652, 1596, 1557, 1536, 1513, 1495, 1455, 1438, 1422, 1411, 1367, 1348, 1303, 1185, 1133, 1092, 1065, 930, 902, 878, 873, 840, 833, 814, 800, 752, 723. HR-MS (ESI); calcd. for $C_{55}H_{59}Br_2N_8O_{10}$ [M+H]$^+$; 1149.2715, found; 1149.2704.

Compound 9

To a stirred solution of Acetyl-5-carboxy-fluorescein (6.2 mg, 14 μmol) in dry DMF (2.0 mL) was added DCC (4.2 mg, 20 μmol) and compound **3-2** (10 mg, 14 μmol) at 0ºC. The reaction mixture was allowed to stir at r.t. for 16 h. After evaporation, the residue was purified by HPLC (ODS-A, flow 9.999 ml/min, gradient; A (CH_3CN containing 0.1% TFA) : B (H_2O containing 0.1% TFA) = 30 : 70 (0 min) to 80 : 20 (50 min)) and the obtained solution was lyophilized to yield compound **9** (2.7 mg, 2.3 μmol, 17%) as white solid. ^{1}H NMR (CD_3OD, 400 MHz, r.t.) δ/ppm = 1.43 (m, 2H), 1.54-1.83 (m, 8H), 2.26 (t, J = 7.2 Hz, 2H), 2.31 (s, 6H), 3.21 (t, J = 6.8 Hz, 2H), 3.39 (m, 2H), 3.66 (s, 2H), 3.80 (s, 6H), 3.92 (t, J = 6.0 Hz, 2H), 6.56 (s, 2H), 6.93-7.00 (m, 4H), 7.22-7.24 (m, 3H), 7.53 (d, J = 8.0 Hz, 1H), 8.20 (s, 2H), 8.59 (dd, J = 8.0, 1.2 Hz, 1H) , 8.85 (br, 1H). ^{13}C NMR (CD_3OD, 100 MHz, r.t.) δ/ppm = 20.99, 23.74, 23.85, 25.36, 30.62, 30.72, 34.87, 36.95, 40.23, 41.26, 56.77, 74.08, 83.62, 107.44, 111.78, 111.88, 116.84, 119.00, 126.53, 128.58, 130.16, 130.28, 131.83, 132.88, 133.73, 137.38, 138.41, 140.48, 149.74, 152.78, 154.17, 155.12, 155.23, 156.25, 159.42, 162.77, 166.30, 170.49, 170.60, 176.23. IR (film): ν (cm^{-1}) 3344, 2951, 2942, 2928, 2923, 1750, 1635, 1559, 1492, 1457, 1419, 1369, 1323, 1291, 1202, 1191, 1149, 1126, 1109, 1075, 1067, 1044, 1033, 1016. HR-MS (ESI); calcd. for $C_{55}H_{53}Br_2N_6O_{14}$ [M+H]$^+$; 1179.1981, found; 1179.1977.

Compound 9-2

To a stirred solution of 5-carboxy-fluorescein (5.1 mg, 14 μmol) in dry DMF (2.0 mL) was added DCC (4.2 mg, 20 μmol), DMAP (0.5 mg, 4 μmol) and compound **3-2** (10 mg, 14 μmol) at 0ºC. The reaction mixture was allowed to stir at r.t. for 17 h. After evaporation, the residue was purified by HPLC (ODS-A, flow 9.999 ml/min, gradient; A (CH_3CN containing 0.1% TFA) : B (H_2O containing 0.1% TFA) = 30 : 70 (0 min) to 80 : 20 (50 min)) and the obtained solution was lyophilized to yield compound **9-2** (5.0 mg, 4.6 μmol, 34%) as white solid. ^{1}H NMR (CD_3OD, 400 MHz, r.t.) δ/ppm = 1.43 (m, 2H), 1.54-1.83 (m, 8H), 2.26 (t, J = 7.2 Hz, 2H), 3.21 (t, J = 6.8 Hz, 2H), 3.39 (m, 2H), 3.66 (s, 2H), 3.80 (s, 6H), 3.92 (t, J = 6.0 Hz, 2H), 6.56 (s, 2H), 6.59 (dd, J = 8.8, 2.4 Hz, 2H), 6.68 (d, J = 8.8 Hz, 2H), 6.73 (d, J = 2.4 Hz, 2H), 7.22 (s, 1H), 7.48 (d, J = 8.0 Hz, 1H), 8.20 (s, 2H), 8.58 (dd, J = 8.0, 1.6 Hz, 1H) , 8.81 (br, 1H). ^{13}C NMR (CD_3OD, 100 MHz, r.t.) δ/ppm = 23.75, 24.14, 25.27, 30.27, 30.62, 34.76, 36.84, 40.12, 41.14, 56.67, 73.98, 79.46, 103.67, 107.33, 109.89, 111.10, 118.31, 118.94, 128.77, 130.25, 131.29, 132.75, 133.64, 133.80, 137.27, 139.81, 140.36, 148.84, 150.09, 155.13, 157.95, 163.63, 165.53, 167.01, 167.79, 171.62, 174.55. IR (film): ν (cm^{-1}) 3300, 2934, 2858, 2853, 1752, 1639, 1553, 1506, 1452, 1376, 1324, 1204, 1143, 1128, 1068, 1043, 1034, 1021, 1014. HR-MS (ESI); calcd. for $C_{51}H_{49}Br_2N_6O_{12}$ [M+H]$^+$; 1095.1770, found; 1095.1753.

Synthesis of LD-BB reagents 10 and 11 for hCA labeling.

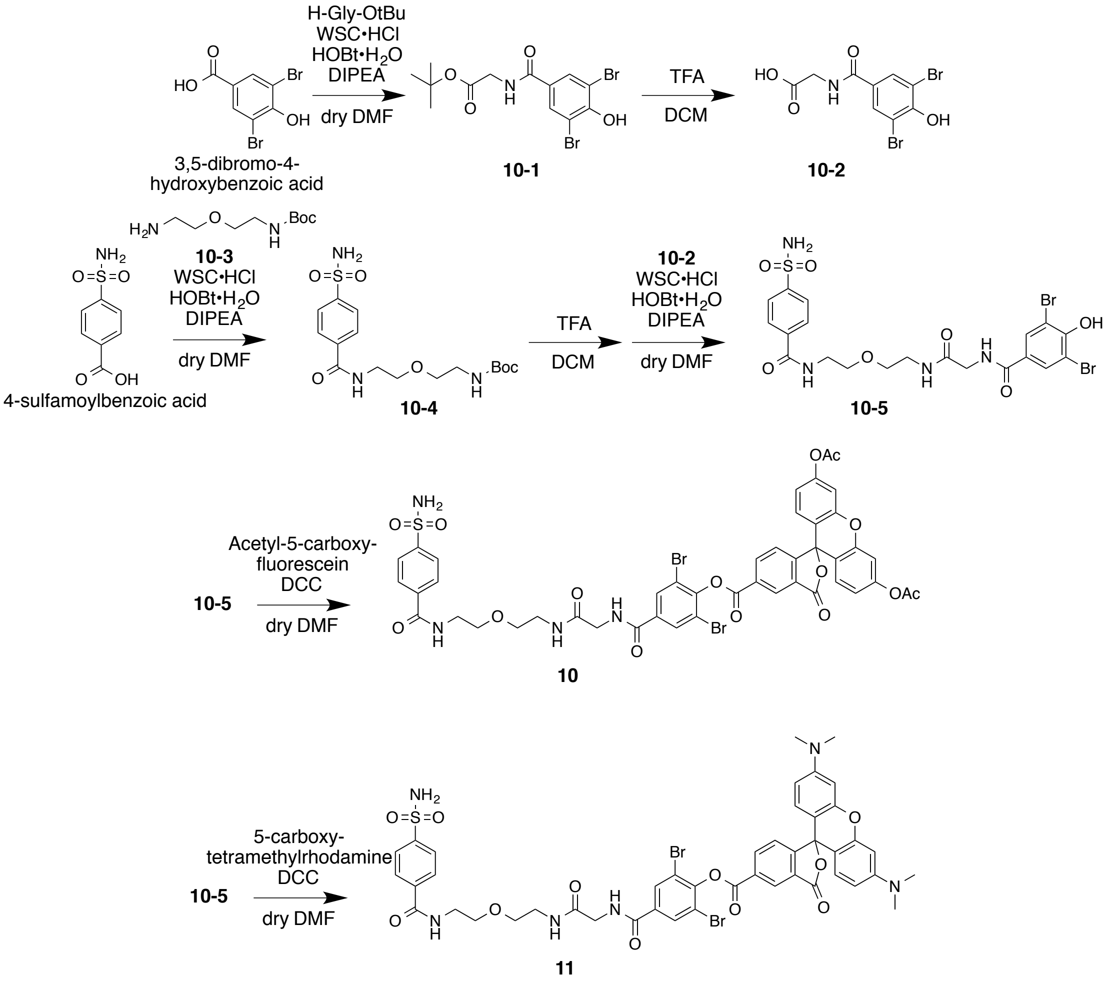

Scheme S8. Synthetic scheme of compound **10** and **11**.

Compound 10-1

To a stirred solution of H-Gly-OtBu (680 mg, 4.1 mmol) in dry DMF (12 mL) was added 3,5-dibromo-4-hydroxybenzoic acid (1.0 g, 3.4 mmol), WSC•HCl (779 mg, 4.1 mmol), HOBt•H_2O (622 mg, 4.1 mmol) and DIPEA (1.75 mL, 10 mmol). The reaction mixture was allowed to stir at r.t. for 12 h. After evaporated, the residue was dissolved in AcOEt (200 mL) and washed with 5% citric acid aq. (120 mL, 3 times), H_2O (120 mL, twice) and brine (120 mL, once). The organic layer was dried over $MgSO_4$, evaporated and the residue was purified by column chromatography on silica gel ($CHCl_3$: MeOH = 100 : 1) to yield compound **10-1** (772 mg, 1.9 mmol, 56%) as white solid. ^{1}H NMR (CD_3OD, 400 MHz, r.t.) δ/ppm = 1.48 (s, 9H), 3.96 (s, 2H), 8.02 (s, 2H). ^{13}C NMR (CD_3OD, 100 MHz, r.t.) δ/ppm = 28.28, 43.30, 82.94, 111.87,

128.62, 132.85, 155.55, 167.43, 170.57. IR (film): ν (cm^{-1}) 3317, 3084, 2982, 2933, 1729, 1645, 1592, 1547, 1474, 1394, 1368, 1303, 1238, 1156, 1081, 1045, 1013, 842, 752. HR-MS (ESI); calc. for $C_{13}H_{15}Br_2N_1O_4Na_1$ $[M+Na]^+$; 429.9260, Found; 429.9254.

Compound 10-2

To a stirred solution of compound **10-1** (88 mg, 220 μmol) in DCM (2.0 mL) was added TFA (2.0 mL). The reaction was monitored by TLC (silica, $CHCl_3$: MeOH = 2 : 1) to confirm the end of this reaction (over 3 h at r.t.). After co-evaporated with toluene (2.0 mL) twice and then dried in vacuo to yield compound **10-2** (77 mg, quantitative) as white solid. ^1H NMR (CD_3OD, 400 MHz, r.t.) δ/ppm = 4.05 (s, 2H), 8.04 (s, 2H). ^{13}C NMR (CD_3OD, 100 MHz, r.t.) δ/ppm = 42.26, 111.84, 128.62, 132.88, 155.51, 167.41, 173.09. IR (film): ν (cm^{-1}) 3313, 3087, 2951, 1728, 1647, 1592, 1549, 1473, 1396, 1305, 1236, 1152, 1016, 900, 769. HR-MS (ESI); calc. for $C_9H_7Br_2N_1O_4Na_1$ $[M+Na]^+$; 373.8634, Found; 373.8630.

Compound 10-4

To a stirred solution of 4-sulfamoylbenzoic acid (591 mg, 2.94 mmol) in dry DMF (13 mL) was added WSC•HCl (704 mg, 3.67 mmol), HOBt•H_2O (565 mg, 3.67 mmol), DIPEA (1.07 mL, 6.12 mmol) and compound **10-3**[S4] (500 mg, 2.45 mmol). The reaction was allowed to stir at r.t. for 13.5 h. After evaporation, the residue was purified by column chromatography on silica gel ($CHCl_3$: MeOH = 25 : 1 to 15 : 1). The obtained compound was dissolved in AcOEt (150 mL), and washed with sat. $NaHCO_3$ aq. (150 mL, twice) and brine (150 mL, once). The organic layer was dried over $MgSO_4$, filtered, evaporated and dried in vacuo to yield compound **10-4** (691 mg, 74%) as white solid. ^1H NMR (CD_3OD, 400 MHz, r.t.) δ/ppm = 1.41 (s, 9H), 3.23 (t, *J* = 5.2 Hz, 2H), 3.52 (t, *J* = 5.2 Hz, 2H), 3.58 (t, *J* = 5.2 Hz, 2H), 3.64 (t, *J* = 7.2 Hz, 2H), 7.97 (m, 4H). ^{13}C NMR (CD_3OD, 100 MHz, r.t.) δ/ppm = 28.73, 41.05, 42.29, 70.32, 70.96, 127.31, 129.04, 139.02, 147.71, 158.54, 168.99. IR (film): ν (cm^{-1}) 3322, 3088, 2978, 2938, 2874, 1691, 1648, 1602, 1542, 1490, 1455, 1393, 1367, 1356, 1290, 1254, 1169, 1124, 1099, 1025, 915, 856, 764, 736, 612. HR-MS (ESI); calc. for $C_{16}H_{25}N_3O_6S_1Na_1$ $[M+Na]^+$; 410.1356, Found; 410.1352.

Compound 10-5

To a stirred solution of compound **10-4** (29 mg, 75 μmol) in DCM (1.0 mL) was added TFA (1.0 mL). The reaction was monitored by TLC (silica, $CHCl_3$: MeOH = 8 : 1) to confirm the end of this reaction (over 2.5 h at r.t.). After co-evaporated with toluene (1.0 mL) twice and then dried in vacuo to yield the target compound (30 mg, quantitative) as white solid.

To a stirred solution of the above compound (30 mg, 75 μmol) in dry DMF (4.0 mL) was added WSC•HCl (21 mg, 110 μmol), HOBt•H_2O (17 mg, 110 μmol), DIPEA (39 μL, 220 μmol) and compound **10-2** (26 mg, 75 μmol).The reaction mixture was allowed to stir at r.t. for 12 h. After evaporation, the residue was purified by column chromatography on silica gel ($CHCl_3$: MeOH = 8 : 1) to yield compound **10-5** (29 mg, 46 μmol, 61%) as yellow oil. ^{1}H NMR (CD_3OD, 400 MHz, r.t.) *δ*/ppm = 3.41 (m, 2H), 3.58 (m, 4H), 3.65 (t, *J* = 5.6 Hz, 2H), 3.97 (s, 2H), 7.97 (s, 4H), 8.04 (s, 2H). HR-MS (ESI); calc. for $C_{20}H_{22}Br_2N_4O_7S_1Na_1$ $[M+Na]^+$; 642.9468, Found; 642.9463. This intermediate compound was used for next step without further characterization except for ^{1}H NMR and HR-MS.

Compound 10

To a stirred solution of Acetyl-5-carboxy-fluorescein (2.2 mg, 4.8 μmol) in dry DMF (1.0 mL) was added DCC 1.5 mg, 7.2 μmol) and compound **10-5** (3.0 mg, 4.8 μmol) at 0ºC. The reaction mixture was allowed to stir at r.t. for 3 h. After evaporation, the residue was purified by HPLC (ODS-A, flow 9.999 ml/min, gradient; A (CH_3CN containing 0.1% TFA) : B (H_2O containing 0.1% TFA) = 30 : 70 (0 min) to 80 : 20 (50 min)) and the obtained solution was lyophilized to yield compound **10** (4.0 mg, 3.8 μmol, 78%) as white solid. ^{1}H NMR (CD_3CN, 400 MHz, r.t.) *δ*/ppm = 2.30 (s, 6H), 3.80 (q, *J* = 5.6 Hz, 2H), 3.55 (m, 4H), 3.64 (t, *J* = 4.8 Hz, 2H), 3.93 (d, *J* = 5.6 Hz, 2H), 5.84 (s, 2H), 6.92 (dd, *J* = 8.8, 2.4 Hz, 2H), 7.00 (m, 3H), 7.19 (d, *J* = 2.4 Hz, 2H), 7.55 (m, 2H), 7.80 (br, 1H), 7.94 (m, 2H), 8.00 (m, 2H), 8.19 (s, 2H), 8.58 (dd, *J* = 8.0, 1.6 Hz, 1H) , 8.83 (d, *J* = 1.6 Hz, 1H). ^{13}C NMR (CD_3CN, 100 MHz, r.t.) *δ*/ppm = 21.20, 39.85, 40.67, 44.21, 69.83, 70.01, 82.80, 111.56, 116.51, 119.41, 126.15, 127.08, 127.12, 128.19, 128.49, 128.68, 128.92, 128.96, 130.19, 131.07, 132.75, 135.68, 138.08, 139.04, 146.45, 149.24, 152.35, 153.71, 158.67, 162.68,

165.08, 167.08, 168.37, 170.01. IR (film): ν (cm^{-1}) 3212, 3044, 2943, 2875, 1759, 1655, 1610, 1554, 1495, 1452, 1424, 1371, 1329, 1290, 1202, 1156, 1129, 1113, 1065, 1047, 1015, 996, 968, 951, 934, 926, 898, 885, 853, 840, 821, 801, 787, 753. HR-MS (ESI); calcd. for $C_{45}H_{36}Br_2N_4O_{15}S_1Na_1$ $[M+Na]^+$; 1085.0157, found; 1085.0189.

Compound 11

To a stirred solution of 5-carboxy-tetramethylrhodamine (2.1 mg, 4.8 μmol) in dry DMF (1.0 mL) was added DCC 1.5 mg, 7.2 μmol) and compound **10-5** (3.0 mg, 4.8 μmol) at 0ºC. The reaction mixture was allowed to stir at r.t. for 3 h. After evaporation, the residue was purified by HPLC (ODS-A, flow 9.999 ml/min, gradient; A (CH_3CN containing 0.1% TFA) : B (H_2O containing 0.1% TFA) = 30 : 70 (0 min) to 80 : 20 (50 min)) and the obtained solution was lyophilized to yield compound **11** (0.8 mg, 0.82 μmol, 17%) as purple solid. ^1H NMR (CD_3OD, 400 MHz, r.t.) δ/ppm = 3.33 (s, 12H), 3.45 (t, J = 5.2 Hz, 2H), 3.61 (m, 4H), 3.67 (t, J = 4.8 Hz, 2H), 4.04 (m, 2H), 7.02 (d, J = 2.4 Hz, 2H), 7.10 (dd, J = 9.6, 2.4 Hz, 2H), 7.20 (d, J = 9.6 Hz, 2H), 7.72 (d, J = 8.0 Hz, 1H), 7.98 (m, 4H), 8.27 (s, 2H), 8.67 (dd, J = 8.0, 2.0 Hz, 1H), 9.11 (br, 1H). HR-MS (ESI); calcd. for $C_{45}H_{43}Br_2N_6O_{11}S$ $[M+H]^+$; 1033.1072, found; 1035.1042.

References

S1) T. Ando, S. Tsukiji, T. Tanaka, T. Nagamune, *Chem. Commun.* 2007, **43**, 4995.

S2) H. Wakabayashi, M. Miyagawa, Y. Koshi, Y. Takaoka, S. Tsukiji, I. Hamachi, *Chem. Asian J.* 2008, **3**, 1134.

S3) K. Matsuo, Y. Kioi, R. Yasui, Y. Takaoka, T. Miki, S. Fujishima and I. Hamachi, *Chem. Sci.* 2013, **4**, 2573-2580.

S4) S.-Y. Han, S. H. Choi, M. H. Kim, W. G. Lee, S. H. Kim, Y. K. Min, B. T. Kim, *Tetrahedron Lett.* 2006, **47**, 2915.
